# A novel IgE epitope-specific antibodies-based sandwich ELISA for sensitive measurement of immunoreactivity changes of peanut allergen Ara h 2 in processed foods

**DOI:** 10.3389/fnut.2024.1323553

**Published:** 2024-02-19

**Authors:** Yan Yan, Liming Li, Caiyun Long, Yaping Dong, Jinyu Li, Caiyi Shen, Yiqian Zhao, Jiangqiang Zhao, Jianbin Wang, Anqi Xiong, Xin Li, Hongbing Chen, Shengfa He

**Affiliations:** ^1^School of Public Health and Health Management, Gannan Medical University, Ganzhou, China; ^2^Department of Dermatology, First Affiliated Hospital of Gannan Medical University, Ganzhou, China; ^3^Department of Laboratory, Ganzhou Center for Disease Control and Prevention, Ganzhou, China; ^4^State Key Laboratory of Food Science and Resources, Nanchang University, Nanchang, China; ^5^Key Laboratory of Environment and Health of Ganzhou, Gannan Medical University, Ganzhou, China

**Keywords:** peanut allergen, Ara h 2, IgE epitope-specific antibodies, sandwich ELISA, IgE-binding

## Abstract

**Background:**

Peanut is an important source of dietary protein for human beings, but it is also recognized as one of the eight major food allergens. Binding of IgE antibodies to specific epitopes in peanut allergens plays important roles in initiating peanut-allergic reactions, and Ara h 2 is widely considered as the most potent peanut allergen and the best predictor of peanut allergy. Therefore, Ara h 2 IgE epitopes can serve as useful biomarkers for prediction of IgE-binding variations of Ara h 2 and peanut in foods. This study aimed to develop and validate an IgE epitope-specific antibodies (IgE-EsAbs)-based sandwich ELISA (sELISA) for detection of Ara h 2 and measurement of Ara h 2 IgE-immunoreactivity changes in foods.

**Methods:**

DEAE-Sepharose Fast Flow anion-exchange chromatography combining with SDS-PAGE gel extraction were applied to purify Ara h 2 from raw peanut. Hybridoma and epitope vaccine techniques were employed to generate a monoclonal antibody against a major IgE epitope of Ara h 2 and a polyclonal antibody against 12 IgE epitopes of Ara h 2, respectively. ELISA was carried out to evaluate the target binding and specificity of the generated IgE-EsAbs. Subsequently, IgE-EsAbs-based sELISA was developed to detect Ara h 2 and its allergenic residues in food samples. The IgE-binding capacity of Ara h 2 and peanut in foods was determined by competitive ELISA. The dose-effect relationship between the Ara h 2 IgE epitope content and Ara h 2 (or peanut) IgE-binding ability was further established to validate the reliability of the developed sELISA in measuring IgE-binding variations of Ara h 2 and peanut in foods.

**Results:**

The obtained Ara h 2 had a purity of 94.44%. Antibody characterization revealed that the IgE-EsAbs recognized the target IgE epitope(s) of Ara h 2 and exhibited high specificity. Accordingly, an IgE-EsAbs-based sELISA using these antibodies was able to detect Ara h 2 and its allergenic residues in food samples, with high sensitivity (a limit of detection of 0.98 ng/mL), accuracy (a mean bias of 0.88%), precision (relative standard deviation < 16.50%), specificity, and recovery (an average recovery of 98.28%). Moreover, the developed sELISA could predict IgE-binding variations of Ara h 2 and peanut in foods, as verified by using sera IgE derived from peanut-allergic individuals.

**Conclusion:**

This novel immunoassay could be a user-friendly method to monitor low level of Ara h 2 and to preliminary predict *in vitro* potential allergenicity of Ara h 2 and peanut in processed foods.

## 1 Introduction

Food allergy is a growing global health concern, affecting up to 10% of the general population (1). One of the most common and severe food allergies is peanut (*Arachis hypogaea*) allergy, an immunoglobulin E (IgE)-mediated food allergy with a prevalence of 1%−3% in developed countries (2). Peanut allergy tends to be lifelong and sub-milligram levels of peanut protein can elicit objective reactions in the most sensitive patients (3). Since there is currently no approved curative treatment for this condition, complete avoidance of peanut proteins is the standard of care. This, however, is often difficult to achieve given the widespread use of peanut as food ingredient and maybe absence of detectable peanut in foods labeled with precautionary (advisory) allergen labeling statements for peanut (4, 5). In addition, peanut allergenicity mainly depends on its IgE epitopes. In the last decade, food processing is increasingly recognized as a method to enhance food tolerance, but the effect of food processing on the structure and allergenicity of peanut proteins is highly variable and therefore difficult to predict (6). Therefore, reliable methods to detect peanut allergenic epitopes and measure changes in IgE-binding ability of peanut in processed foods are warranted.

Analytical methods currently used to detect peanut allergens, such as real-time polymerase chain reaction (7), reversed-phase high-performance liquid chromatography (RP-HPLC) (8), liquid chromatography coupled mass spectrometry (9), enzyme-linked immunosorbent assay (ELISA) (10–12), and lateral flow immunoassay (10), lack the ability to specifically detect allergenic epitopes of the allergens. Traditionally, the method for measurement of IgE-binding capacity variations of peanut allergens is based on patients' IgE antibodies (13–15). However, the limited and variable sera from peanut-allergic patients makes the standardization of the detection method very difficult for commercial purposes. Hence, there is a need for more efficient and simplistic analytical methods that detect minute traces of peanut allergens and reveal changes in the IgE-immunoreactivity of peanut allergens in foods.

One of the analytical methods that can be used for allergen detection and is characterized by high specificity and sensitivity, low cost, and simplicity is ELISA. Recently, an ELISA based on IgE epitope-specific antibodies (IgE-EsAbs) was successfully used for the prediction of IgE-immunoreactivity variations of milk in food samples (16). This technique aims to detect specific IgE epitopes in the allergen, which play vital roles in triggering the allergic cascade and hence may be used to preliminary predict *in vitro* food potential allergenicity (17, 18). One of the most widely characterized allergens in peanut is Ara h 2, which is shown to be the most potent allergen and the best predictor of peanut allergy (19, 20). Therefore, IgE epitopes in Ara h 2 could serve as reliable biomarkers for measurement of potential changes in IgE-immunoreactivity of Ara h 2 in foods. Based on this, we hypothesized that an ELISA based on IgE-EsAbs directed against Ara h 2 could be used to accurately detect the IgE epitope content of Ara h 2, thereby revealing the IgE-binding changes of Ara h 2 and peanut in processed foods in a cost-efficient and simplistic manner (18).

**Figure 1:**
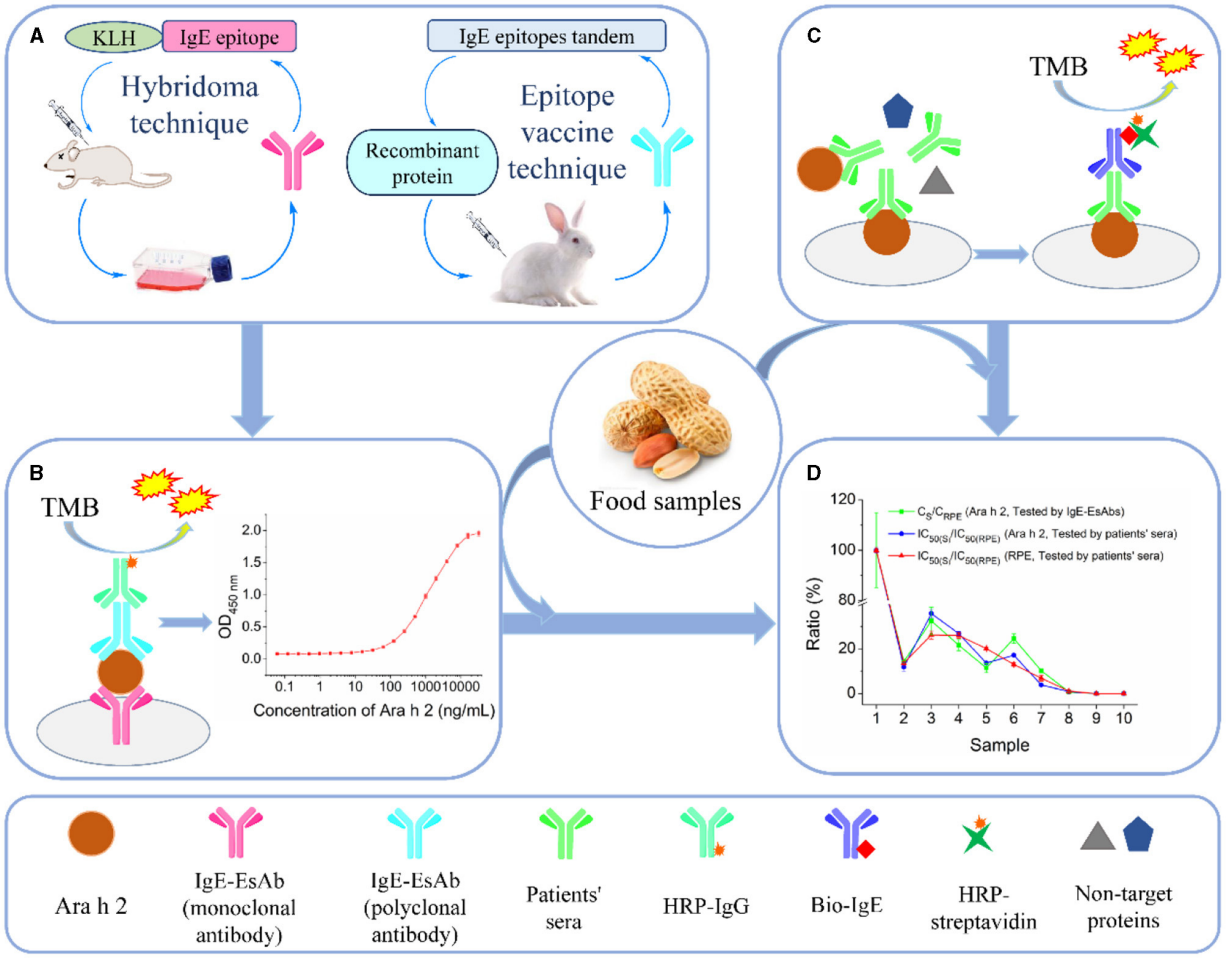
Schematic illustration of the development and validation of the IgE-EsAb-based sELISA for detection of Ara h 2 and prediction of peanut IgE-immunoreactivity in foods. **(A)** Generation of monoclonal and polyclonal antibodies specifically against IgE epitope(s) of Ara h 2 for use as capture and detection antibodies in the immunoassay, respectively. **(B)** Schematic representation of the IgE-EsAb-based sELISA approach for detection of Ara h 2. **(C)** Assessment of IgE-binding capacity in food samples using sera IgE from peanut-allergic individuals for use in assay validation. **(D)** Assay validation by comparing the results obtained using the IgE-EsAb-based sELISA with those obtained using sera IgE. Results compared are the relationship between Ara h 2 IgE-binding ability and peanut IgE-binding ability, and the dose-effect relationship between the Ara h 2 IgE epitope content and Ara h 2 (or peanut) IgE-binding ability.

In this study, our objective was to develop an IgE-EsAbs-based sandwich ELISA (sELISA) for detecting allergenic residues of Ara h 2 and evidencing changes in the IgE-immunoreactivity of Ara h 2 in foods (Figure 1). Briefly, a monoclonal antibody against the major IgE epitope of Ara h 2 and a polyclonal antibody against twelve IgE epitopes of Ara h 2 were generated for use as capture and detection antibodies in the assay (Figures 1A, B). Next, the IgE-EsAbs-based sELISA was used to detect Ara h 2 and its allergenic residues in food samples, and results were compared to those obtained using sera IgE derived from peanut-allergic individuals (Figures 1C, D).

## 2 Materials and methods

### 2.1 Materials and reagents

DEAE Sepharose Fast Flow, Histrap^TM^ HP affinity column (1 mL), and HiTrap^TM^ Protein A HP affinity column (1 mL) were purchased from GE Healthcare (Uppsala, Sweden). Prestained protein marker and 3,3',5,5'-Tetramethylbenzidine (TMB) were obtained from Thermo Fisher Scientific (Rockford, USA). Complete Freund's adjuvant, incomplete Freund's adjuvant, gelatin from cold water fish, α-lactalbumin, β-lactoglobulin, casein, goat anti-rabbit HRP-IgG, rabbit anti-mouse HRP-IgG, and biotin-labeled goat anti-human IgE (Bio-IgE) were purchased from Sigma (St. Louis, USA). IgE epitope peptides (purity ≥ 95%, RP-HPLC) were synthesized by GL Biochem (Shanghai, China). Food samples were purchased from local supermarkets. Peanut allergy patients' sera were provided by the First Affiliated Hospital of Gannan Medical University and approve by Gannan Medical University Research Ethics Committee (Reference number 2021105, 8/March/2021), details of which are shown in Supplementary Table S1. All reagents were analytical grade and solutions were prepared using ultra-pure water throughout the experiments.

### 2.2 Purification of peanut allergen Ara h 2

Ara h 2 was isolated from raw peanut protein extract according to the methods described in Hu et al. (21), with minor modifications. Briefly, raw peanut seeds were ground into peanut butter and defatted three times with acetone containing 0.07% β-mercaptoethanol at a 1:5 (w/v) ratio while being stirred at 25°C for 2 h. After centrifugation (12,000 × g for 10 min at 4°C), the precipitate was collected and air-dried. Next, the protein from the defatted powder (20.0 g) was extracted by addition of 100 mL Tris-HCl buffer (50 mmol/L, pH 7.2), followed by incubation at 25°C for 2 h while stirring. After centrifugation, the supernatant (peanut protein extract) was collected and Ara h 2 was subsequently isolated from the supernatant by DEAE Sepharose Fast Flow anion exchange chromatography followed by sodium dodecyl sulfate polyacrylamide gel electrophoresis (SDS-PAGE). Shortly, the chromatographic column (1.6 cm × 50 cm) was equilibrated with Tris-HCl (50 mmol/L, pH 7.2) and subsequently loaded with 10 mL peanut protein extract, after which the loaded column was washed with equilibrating buffer containing 0.04 mol/L NaCl. The proteins were further eluted using 600 mL of 0.04–0.2 mol/L NaCl gradient in equilibrating buffer. After dialysis and lyophilization of the collected eluates, the eluates were subjected to SDS-PAGE and the Ara h 2 fraction was excised from the SDS-PAGE gel. The purity of Ara h 2 was analyzed by ImageJ software.

### 2.3 Generation of a monoclonal antibody against IgE epitope of Ara h 2

A mouse monoclonal antibody (mAb, 2K9-1) against the peptide sequence NH2-DRRCQSQLER-COOH (B3), selected based on the sequence of the most dominant IgE epitope of Ara h 2 (22, 23), was prepared by Abmart (Shanghai, China) and used as a capture antibody in the IgE-EsAb-based sELISA.

### 2.4 Generation of a polyclonal antibody against IgE epitopes of Ara h 2

IgE-EsAbs for use as detection antibody in the IgE-EsAb-based sELISA were obtained following inoculation of rabbits with a multiepitope-based vaccine (a recombinant protein), comprising of a T cell epitope, IgE epitopes, and linkers, as detailed below.

#### 2.4.1 Construction of an expression system for recombinant tAra h 2

A tandem containing twelve IgE-binding epitopes of Ara h 2 (tAra h 2) was designed as described previously (24). In short, twelve IgE epitopes of Ara h 2 (B1-B12) were selected as part of the tandem based on Stanley et al. (22) and Shreffler et al. (25) epitope mapping results, and one dominant T cell epitope (AA94–113) of Ara h 2 was selected (26–28). The epitope sequences are shown in Supplementary Table S2. To construct the tAra h 2, the T cell epitope and B1–B12 were situated on the N-terminal and C-terminal respectively, and four glycines (GGGG) were inserted as a linker between two adjacent epitopes. Next, the gene sequence of tAra h 2 was custom-synthesized and cloned into the pET-28a(+) expression plasmid. Following confirmation of successful cloning by DNA sequencing, the plasmids were transformed into *E. coli* BL21 (DE3) pLysS cells by Chinapeptides (Shanghai, China).

#### 2.4.2 Expression and purification of recombinant tAra h 2

Expression of recombinant tAra h 2 by *E. coli* BL21 (DE3) pLysS cells was induced by incubating the cells with 0.6 mmol/L isopropyl-β-d-thiogalactoside (IPTG) at OD_600__nm_ ~0.6 at 26°C for 4 h. After centrifugation (12,000 × g for 10 min at 4°C), the cell pellet was resuspended in 10 mmol/L phosphate buffer saline (PBS, pH 7.2) and cells were subsequently lysed by ultrasonication. After centrifugating again, the recombinant tAra h 2 in the supernatant was purified by Histrap^TM^ HP according to the manufacturer's instructions, and the purity of recombinant tAra h 2 was analyzed by ImageJ software.

#### 2.4.3 Production and purification of a tAra h 2-specific polyclonal antibody

The animal study was approved by Gannan Medical University Animal Care Committee, under the guidelines of China Council for Animal Care (SYXK-Gan 2018-0004, China). Two8-week-old male New Zealand white rabbits were purchased from the Ganzhou Institute of Animal Husbandry (SCXK-Gan 2018-0009, China). After collecting the negative serum from auricular vein, the rabbits were subcutaneously immunized with 1 mg recombinant tAra h 2 (2 mg/mL) emulsified with complete Freund's adjuvant in a total volume of 1 mL as a priming dose. Subsequently, the rabbits received three 1 mL booster injections containing the same dose of antigen emulsified in incomplete Freund's adjuvant in 2-week intervals for the production of tAra h 2-specific polyclonal antibody (pAb-tAra h 2). One week after the last immunization, blood samples were taken from the carotid artery and were clotted overnight at 4°C. The serum was isolated by centrifugation at 4,500 × g for 10 min at 4°C. Then, the IgG (pAb-tAra h 2) was purified by HiTrap^TM^ Protein A HP according to the manufacturer's instructions, and the obtained pAb-tAra h 2 was stored at −80°C until use.

### 2.5 Characterization of the generated monoclonal and polyclonal antibodies

Target binding and specificity of the generated monoclonal and polyclonal antibodies for use in the IgE-EsAb-based sELISA were evaluated as detailed below.

#### 2.5.1 Analysis of affinity constant of monoclonal antibody

The affinity constant (*K*_*aff*_) of 2K9-1 to Ara h 2 was analyzed with indirect ELISA as described previously (29). Briefly, a microliter plate was pre-coated overnight at 4°C with three different concentrations of Ara h 2 (0.5 μg/mL, 1 μg/mL, and 2 μg/mL), after which wells were washed three times with PBS containing 0.05% Tween-20 (PBST). Next, wells were blocked with 3% gelatin in PBS for 1 h at 37°C. After washing, serial concentrations (2,000 ng/mL, 1,000 ng/mL, 500 ng/mL, 250 ng/mL, 125 ng/mL, 62.5 ng/mL, 31.25 ng/mL, and 15.625 ng/mL) of 2K9-1 was added and incubated for 1 h at 37°C. The wells were washed and subsequently incubated with 100 μL of rabbit anti-mouse HRP-IgG (diluted 1:10,000 in PBS) for 1 h at 37°C. After washing again, 100 μL of TMB substrate for HRP was added and incubated for 15 min at 37°C, followed by addition of 50 μL of 2 mol/L sulfuric acid and immediate measurement of optical density at 450 nm (OD_450nm_) using a microplate reader (Varioskan LUX; Thermo Fisher Scientific, USA). The *K*_*aff*_ of 2K9-1 was calculated as follows: *K*_*aff*_= (n - 1)/2(n[Ab']t - [Ab]t), where n = [Ag]/[Ag'], [Ag] and [Ag'] are two different coating concentrations of Ara h 2, and [Ab]t and [Ab']t are the concentrations (in mol/L) of 2K9-1 at which 50% of the maximum OD_450nm_ values were obtained for plates coated with [Ag] and [Ag'], respectively.

#### 2.5.2 Analysis of the titer of polyclonal antibody

The titers of tAra h 2-specific antibodies in the collected rabbit serum were determined by indirect ELISA. Microplates were coated with 100 μL of recombinant tAra h 2 (1 μg/mL) overnight at 4°C. After washing three times with PBST, each well was blocked with 250 μL of 3% gelatin in PBS for 1 h at 37°C. After washing, a dilution series of rabbit serum (100 μL/well) was added and incubated for 1 h at 37°C. Next, wells were washed and subsequently incubated with 100 μL of goat anti-rabbit HRP-IgG (diluted 1:5,000 in PBS) for 1 h at 37°C. After washing, wells were incubated with 100 μL of TMB solution for 15 min at 37°C, after which 50 μL of sulfuric acid (2 mol/L) was added to stop the color development and the OD_450nm_ was measured using a microplate reader.

The serum antibody titer was defined as the maximum dilution factor that yielded P/N > 2.1, and P > 0.2 (*n* = 3), in which P and N represent the OD_450nm_ of positive and negative serum, respectively.

#### 2.5.3 Evaluation of antibody binding to IgE epitope(s) of Ara h 2

Binding of 2K9-1 and pAb-tAra h 2 to the target IgE epitope(s) of Ara h 2 was assessed by competitive ELISA (cELISA), as described previously (30). In short, the plates were coated with 100 μL of purified Ara h 2 (0.25 μg/mL) overnight at 4°C. After washing and blocking, wells were incubated with 50 μL of varying concentrations of IgE epitope peptide (0.25, 0.5, or 1 μg/mL for 2K9-1; 0.25, 1, or 4 μg/mL for pAb-tAra h 2;) and 50 μL of a fixed concentration of antibody (31.25 ng/mL for 2K9-1; 2 μg/mL for pAb-tAra h 2) for 1 h at 37°C. After washing, wells were incubated with 100 μL of rabbit anti-mouse HRP-IgG (diluted 1:10,000 in PBS, for 2K9-1) or goat anti-rabbit HRP-IgG (diluted 1:5,000 in PBS, for pAb-tAra h 2) for 1 h at 37°C, and subsequently washed again. Next, wells were incubated with 100 μL TMB solution for 15 min at 37°C, followed by addition of 50 μL of 2 mol/L sulfuric acid and immediate measurement of optical density as detailed above.

#### 2.5.4 Evaluation of antibody specificity

The cross-reactivity (CR) of 2K9-1 and pAb-tAra h 2 with various allergens was analyzed by cELISA. First, protein as a source of allergens was extracted from different foods. Protein from egg, soybean, oat, and wheat were extracted as our previously reported method (29). Protein from cashew, macadamia, pistachio, chestnut, almond, sesame, and walnut were first powdered and subsequently defatted using acetone (1:10, w/v). Proteins were then extracted from 1 g defatted powder addition of 20 mL Tris-HCl (50 mmol/L, pH 8.0, containing 2% Tween-20) and subsequent incubation for 4 h at 25°C while stirring. After centrifugation (12,000 × g for 10 min at 4°C), the supernatant was collected for use in the cELISA.

The competitive concentrations of Ara h 2 and inhibitors (protein extracts) were 2-fold serially diluted from 16 μg/mL to 0.125 μg/mL and 128 μg/mL to 32 μg/mL, respectively. The ELISA procedures were in accordance with the cELISA described above. The 50% inhibition concentration (IC_50_) was used to determine the CR as follows: CR (%) = [IC_50(Arah2)_/IC_50(inhibitor)_] × 100%.

### 2.6 Development of the IgE-EsAbs-based sELISA for Ara h 2 detection

The microtiter plate was coated with 100 μL of 2K9-1 (capture antibody, 1 μg/mL) and incubated overnight at 4°C. After washing three times with PBS containing 0.2% Tween-20 (PBST), the wells were blocked with 250 μL of 3% gelatin in PBST and incubated for 1 h at 37°C. The wells were washed and 100 μL of Ara h 2 (or food samples and blocking buffer as control) was added, followed by incubation for 2 h at 37°C. After washing again, 100 μL of pAb-tAra h 2 (detection antibody, 4 μg/mL) was added and incubated for 1 h at 37°C. After removal of unbound pAb-tAra h 2 by washing, 100 μL of goat anti-rabbit HRP-IgG (diluted 1:5,000) was added to the wells and incubated for 0.5 h at 37°C. After washing, 100 μL of TMB substrate solution was added and color was developed for 20 min at 37°C. Color development was terminated using 50 μL of 2 mol/L sulfuric acid, after which the OD_450nm_ was measured using a microplate reader. To reduce non-specific adsorption, the Ara h 2, food samples, pAb-tAra h 2, and goat anti-rabbit HRP-IgG were diluted with a blocking solution (3% gelatin in PBST).

### 2.7 Evaluation of the sensitivity, accuracy, precision, and specificity of the IgE-EsAbs-based sELISA for Ara h 2 detection

The limit of detection (LOD) and quantitation (LOQ), accuracy, and precision of the developed IgE-EsAbs-based sELISA were estimated using the Eurachem Guidance on validating analytical methods (31). LOD and LOQ were computed as the concentration of Ara h 2 corresponding to the mean of ten blank values plus three or ten standard deviations (SD), respectively. The accuracy was checked by analyzing the bias (%), which was defined as the difference (%) between the Ara h 2 concentration detected by the developed sELISA and the actual concentration of Ara h 2. The precision of the proposed sELISA was assessed by testing the relative SD of repeatability (RSDr, intra-day) and reproducibility (RSDR, inter-day) at a series of Ara h 2 concentrations. Repeatability and reproducibility were determined by analyzing Ara h 2 at different concentrations in 1 day (*n* = 5) and in five different days (*n* = 3), respectively. Results were computed as follows: RSDr or RSDR (%) = SD/mean × 100%.

The specificity of the developed sELISA was evaluated with various proteins (i.e., α-lactalbumin, β-lactoglobulin, casein, and proteins extracted from egg, soybean, wheat, oat, cashew, macadamia, pistachio, chestnut, almond, sesame, and walnut) for CR at 0.125 μg/mL, 0.5 μg/mL, 2.0 μg/mL, and 8.0 μg/mL.

### 2.8 Evaluation of applicability of the IgE-EsAbs-based sELISA

A spike/recovery experiment was performed to investigate the capacity of the IgE-EsAbs-based sELISA to accurately detect Ara h 2 in samples with complex matrices. First, proteins were extracted from different foods. Proteins from boiled peanut, roasted peanut, and fried peanut were extracted as described above for raw peanut. Proteins from cookie, bread, and dry baked cake were extracted by first powdering the food, followed by addition of 20 mL Tris-HCl (50 mmol/L, pH 8.0, containing 2% Tween-20) to the powder (1 g) and agitation for 4 h at 25°C. Samples were then centrifuged (12,000 × g for 10 min at 4°C) and supernatants were collected. Protein extracts from beverages were obtained by centrifugation, followed by collection of supernatants. Protein extracts of peanuts and beverages were spiked with 0, 0.25, or 2.0 mg/mL Ara h 2, and those of cookie, bread, and dry baked cake were spiked with 0, 0.25, or 2.0 mg/g Ara h 2. Samples were analyzed using IgE-EsAbs-based sELISA, and the recovery was calculated as follows: Recovery (%) = (A2–A0)/A1 × 100%, where A0 represents the detected concentration of a sample without spiked Ara h 2, A1 the concentration of Ara h 2 used for spiking, and A2 the detected concentration of a sample spiked with Ara h 2.

### 2.9 Assessment of IgE-binding capacity of food samples

The IgE-binding capacity of Ara h 2 and peanut in food samples was determined by cELISA. The microplate was coated with 100 μL of Ara h 2 or raw peanut extract (RPE) at 2 μg/mL and incubated overnight at 4°C. After washing three times with PBS containing 0.1% Tween-20 (PBST), the wells were blocked with 3% gelatin in PBST and incubated for 1 h at 37°C. After washing, equal volume (50 μL) of food samples and pooled sera (diluted 1:10 for Ara h 2; diluted 1:30 for RPE) were added and incubated for 1 h at 37°C. After washing thrice, 100 μL of Bio-IgE (diluted 1:2500) was added and incubated for 1 h at 37°C. After washing again, 100 μL of HRP-streptavidin (diluted 1:60) was added and incubated for 1 h at 37°C. The subsequent procedures were in accordance with the cELISA described above. To reduce non-specific adsorption, food samples, pooled sera, Bio-IgE, and HRP-streptavidin were diluted in blocking solution (3% gelatin in PBST).

### 2.10 Statistical analysis

Data are reported as mean ± SD. Statistical analyses were performed using SPSS 17.0 (SPSS Inc., Chicago, USA) and statistical significance was assessed using Tukey's pairwise comparisons of ANOVA. Differences were considered significant when ^*^p < 0.05 and ^**^p < 0.01.

## 3 Results and discussion

### 3.1 Purification of Ara h 2

**Figure 2:**
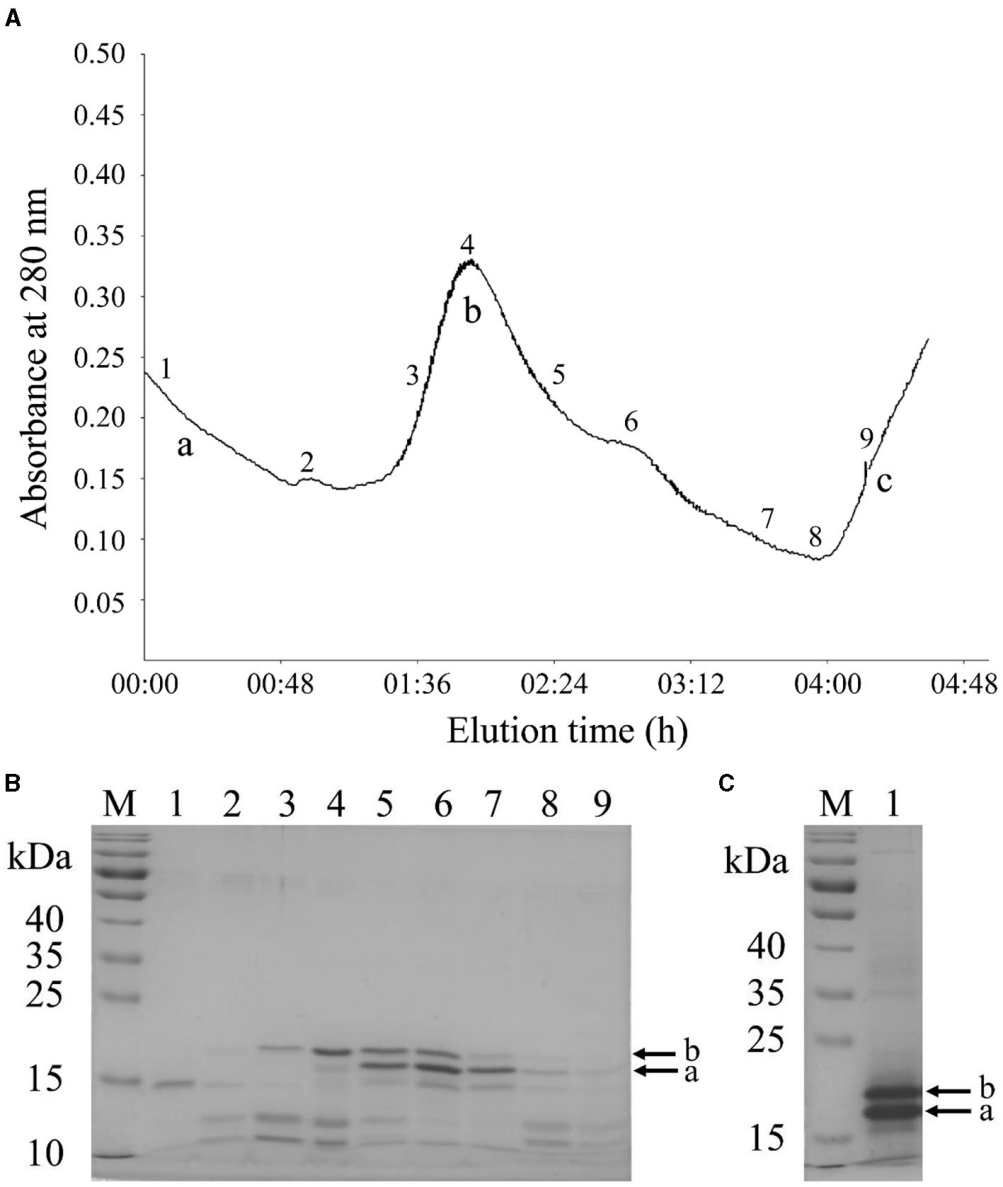
Purification of Ara h 2 by two-step method. **(A)** Chromatogram of raw peanut protein extract using DEAE-Sepharose Fast Flow anion-exchange chromatography. **(B)** SDS-PAGE patterns of Ara h 2 purified by anion-exchange chromatography. M: markers; lanes 1 to 9: fractions of 1 to 9 in anion-exchange chromatography profile. **(C)** SDS-PAGE patterns of isolated Ara h 2 from the SDS-PAGE gel. M: markers; lane 1: purified protein from the gel. The bands of Ara h 2.01 (a) and Ara h 2.02 (b) are indicated by arrows. Letters a–c: three major peaks.

The raw peanut protein extract was fractionated into three major peaks (a, b, and c) using anion exchange chromatography under linear gradient elution (Figure 2A). Then, the eluted fractions were analyzed by SDS-PAGE (Figure 2B). The eluates of peak “b” contained two distinct bands with molecular masses ranging from 18 to 20 kDa (Figure 2B, lanes 3–8), which are corresponding to Ara h 2.01 and Ara h 2.02, respectively (21). The purity of Ara h 2 in the eluates, however, was only between 46.61% and 80.95% (Figure 2B, lanes 4–7) as a result of co-elution of Ara h 6 (15 kDa), which has a high homology with Ara h 2 and therefore has similar physical and chemical properties (20, 32). To improve the purity of Ara h 2, the eluates between positions “4” and “7” in Figure 2A were collected, dialyzed, lyophilized, and subsequently subjected to SDS-PAGE. Ara h 2 protein extracted from the SDS-PAGE gel showed a purity of 94.44% (Figure 2C), and the obtained Ara h 2 was identified by mass spectrometry (Supplementary Figure S1). These results indicate that high purity Ara h 2 was obtained by the employed two-step purification method.

### 3.2 Expression and purification of recombinant tAra h 2

The amino acid and gene sequences of the designed tAra h 2 are shown in Supplementary Figure S2. Sequencing revealed that the constructed expression plasmid pET28a(+)-tAra h 2 contained the full gene sequence of tAra h 2 in expression strain *E. coli* BL21 (DE3) pLysS (Supplementary Figure S3, located 225–725 bp), indicating that the expression strain was successfully constructed.

**Figure 3:**
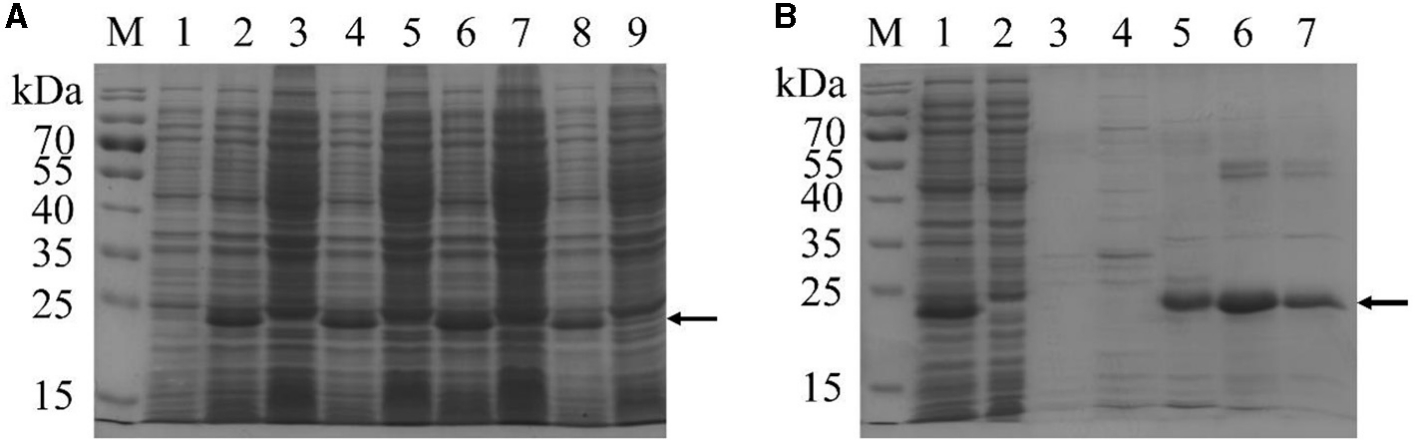
SDS-PAGE analysis of recombinant tAra h 2. **(A)** Expression of recombinant tAra h 2 under different induction conditions. M: markers; lanes 1, 3, 5, 7, and 9: incubation without IPTG for 0 h, 2 h, 3 h, 4 h, and 5 h, respectively; lanes 2, 4, 6, and 8: induction with 0.6 mmol/L IPTG for 2 h, 3 h, 4 h, and 5 h, respectively. **(B)** Purification of recombinant tAra h 2 by Histrap^TM^ HP. M: markers; lane 1: supernatant of *E.coli* lysates after centrifugation; lane 2: flow-through protein of the column; lane 3: non-specific elution with 10 column volumes of 20 mmol/L imidazole in PBS (10 mmol/L, containing 0.5 mol/L NaCl, pH 7.2); lanes 4–7: specific elution with 5 column volumes of 25, 50, 100, and 200 mmol/L imidazole in PBS, respectively. The recombinant tAra h 2 is indicated by arrows.

To test whether recombinant tAra h 2 could be expressed by the expression strain, cells were incubated with 0.6 mmol/L IPTG at 26°C to induce expression. Following induction, a major band with an apparent molecular weight slightly below 25 kDa was observed, particularly after 4 h of induction (Figure 3A). The band presumably corresponding to recombinant tAra h 2 appeared at a greater molecular weight than the expected molecular mass (~18.03 kDa). This phenomenon is consistent with other reported His-tag fusion proteins (33–35). Thus, these results indicate that the recombinant tAra h 2 was successfully expressed.

Following induction of expression by incubation with 0.6 mmol/L IPTG at 26°C for 4 h, the cells were harvested by centrifugation. The pellet was sonicated, and the recombinant tAra h 2 in the supernatant was purified by Histrap^TM^ HP. As shown in Figure 3B, most of the recombinant tAra h 2 was bound to the column after loading the supernatant (lanes 1 and 2), and there was no protein after non-specific elution (lane 3). The His-tagged protein bound to the Histrap^TM^ HP column was eluted using different concentrations of imidazole (Figure 3B, lanes 4–7), and recombinant tAra h 2 was obtained at a purity of 88.56% (Figure 3B, lane 6).

### 3.3 Production and characterization of Ara h 2-specific antibodies for use in the IgE-EsAbs-based sELISA

#### 3.3.1 Immunological characterization of capture antibody 2K9-1

**Figure 4:**
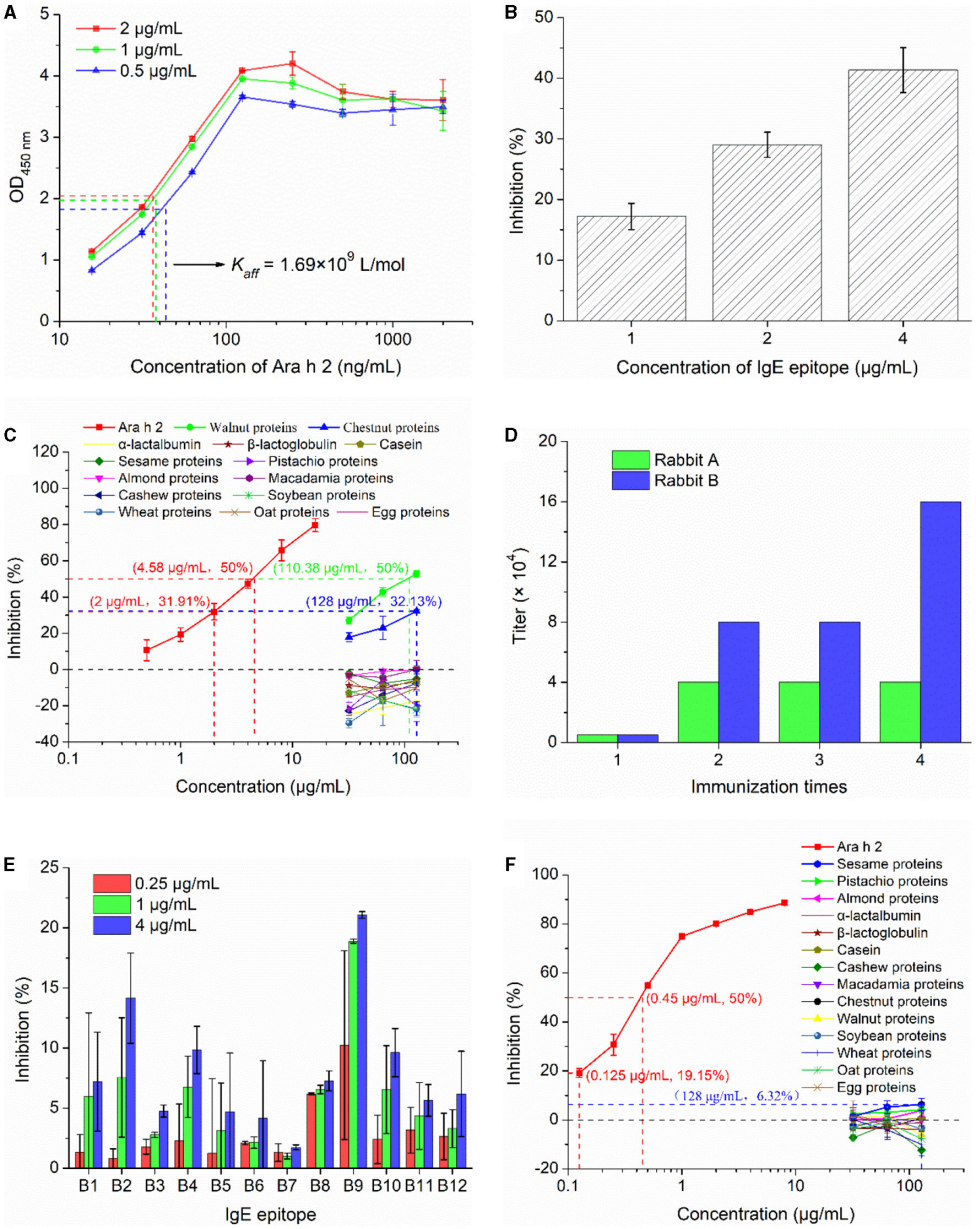
Immunological characterization of mAb 2K9-1 and pAb-tAra h 2. **(A)** Affinity constant of 2K9-1 to Ara h 2. The dash lines are the concentrations of 2K9-1 at 50% of the largest absorbance in the plate coated with different concentrations of Ara h 2. **(B)** Binding ability of 2K9-1 to IgE epitope. **(C)** Cross-reactivity of 2K9-1 with food allergens. **(D)** The titers of antisera against recombinant tAra h 2. **(E)** Binding ability of pAb-tAra h 2 to IgE epitopes (B1–B12). **(F)** Cross-reactivity of pAb-tAra h 2 with food allergens. Data are presented as mean ± SD (n = 3).

The *K*_*aff*_ of mouse monoclonal antibody 2K9-1 against Ara h 2 was analyzed by indirect ELISA. The concentration of 2K9-1 at half of the maximum absorbance in the plate coated with 2, 1, and 0.5 μg/mL of Ara h 2 were 36.37, 37.85, and 43.44 ng/mL, respectively. Consequently, the average *K*_*aff*_ was calculated as 1.69 × 10^9^ L/mol (Figure 4A).

The ability of 2K9-1 to bind to its target IgE epitope (B3) of Ara h 2 was assessed by cELISA. The results show that the inhibition increased with increasing peptide concentration (Figure 4B), indicating that 2K9-1 binds its target IgE epitope of Ara h 2. In addition, the epitope B3 can be recognized by sera IgE from most peanut-allergic patients, and has been identified as the most dominant IgE epitope of Ara h 2 (22, 23). This suggests that this epitope remains stable after processing and gastrointestinal digestion. As a result, this epitope can work as a dependable biomarker, and the prepared 2K9-1 can serve as an efficient tool for detecting Ara h 2 and measuring its IgE-binding changes in foods.

The specificity of 2K9-1 for Ara h 2 was additionally determined by cELISA (Figure 4C). The IC_50_ of Ara h 2 was 4.58 μg/mL. The 2K9-1 showed no binding to cow's milk proteins (α-lactalbumin, β-lactoglobulin, and casein) or to proteins from sesame, pistachio, almond, macadamia, cashew, soybean, wheat, oat, and egg when these proteins at a concentration of 128 μg/mL. However, slight CR was observed with walnut proteins and chestnut proteins. The IC_50_ of walnut proteins was 110.38 μg/mL, corresponding to a CR of 4.15%. For chestnut proteins, an inhibition rate of 32.13% was observed at a concentration of 128 μg/mL. This inhibition rate is similar to that of Ara h 2 at 2 μg/mL (31.91%). Hence, it can be speculated that the CR with chestnut proteins was ~1.56%. These might be due to Ara h 2 sharing a similar IgE-reactive epitope with walnut (Jug r 2) and chestnut allergen (36). These results indicate that the 2K9-1 is highly specific.

#### 3.3.2 Immunological characterization of detection antibody pAb-tAra h 2

For the production of polyclonal antibodies against recombinant tAra h 2 (pAb-tAra h 2), rabbits were inoculated with the purified recombinant tAra h 2 four times. Following inoculation, the titer values of antisera were determined as 40,000 and 160,000 for rabbits A and B (Figure 4D), respectively. Therefore, the serum from rabbit B was selected for the purification of pAb-tAra h 2 using the HiTrap^TM^ Protein A HP column.

As recognition of IgE epitopes of native Ara h 2 by pAb-tAra h 2 is critical for successfully detecting Ara h 2 allergenic residues and measuring potential changes in IgE-immunoreactivity of Ara h 2 in foods (18, 30), the binding of the purified pAb-tAra h 2 to twelve selected IgE epitopes of Ara h 2 was analyzed by cELISA (Figure 4E). The results show that the pAb-tAra h 2 recognized all selected IgE epitopes, and the inhibition increased with increasing epitope peptide concentration. These findings suggest that the content of Ara h 2 IgE epitopes in foods can be detected by pAb-tAra h 2.

The specificity of pAb-tAra h 2 for Ara h 2 were determined by cELISA. The IC_50_ of Ara h 2 was 0.45 μg/mL (Figure 4F). The pAb-tAra h 2 did not show binding to proteins from cow's milk (α-lactalbumin, β-lactoglobulin, and casein), cashew, macadamia, chestnut, walnut, soybean, wheat, oat, and egg at any of the tested protein concentrations (32–128 μg/mL). However, slight inhibition ratio was observed when protein extracted from sesame (6.32%), pistachio (4.26%), and almond (3.82%) at 128 μg/mL. This might be due to Ara h 2 sharing common IgE-binding epitopes with sesame, pistachio, and almond allergens (37, 38). These inhibition rates were significantly (*p* < 0.01) lower than 19.16% when the concentration of Ara h 2 was 0.125 μg/mL, thereby indicating that the CR with sesame, pistachio, and almond was lower than 0.098%. These findings suggest that the pAb-tAra h 2 is highly specific.

### 3.4 Performance evaluation of the IgE-EsAbs-based sELISA

Using the abovementioned Ara h 2-specific capture and detection antibodies, an IgE-EsAbs-based sELISA for Ara h 2 detection was set up and tested for sensitivity, accuracy, precision, and specificity as detailed below.

#### 3.4.1 Sensitivity evaluation and comparative analysis of the IgE-EsAbs-based sELISA

**Figure 5:**
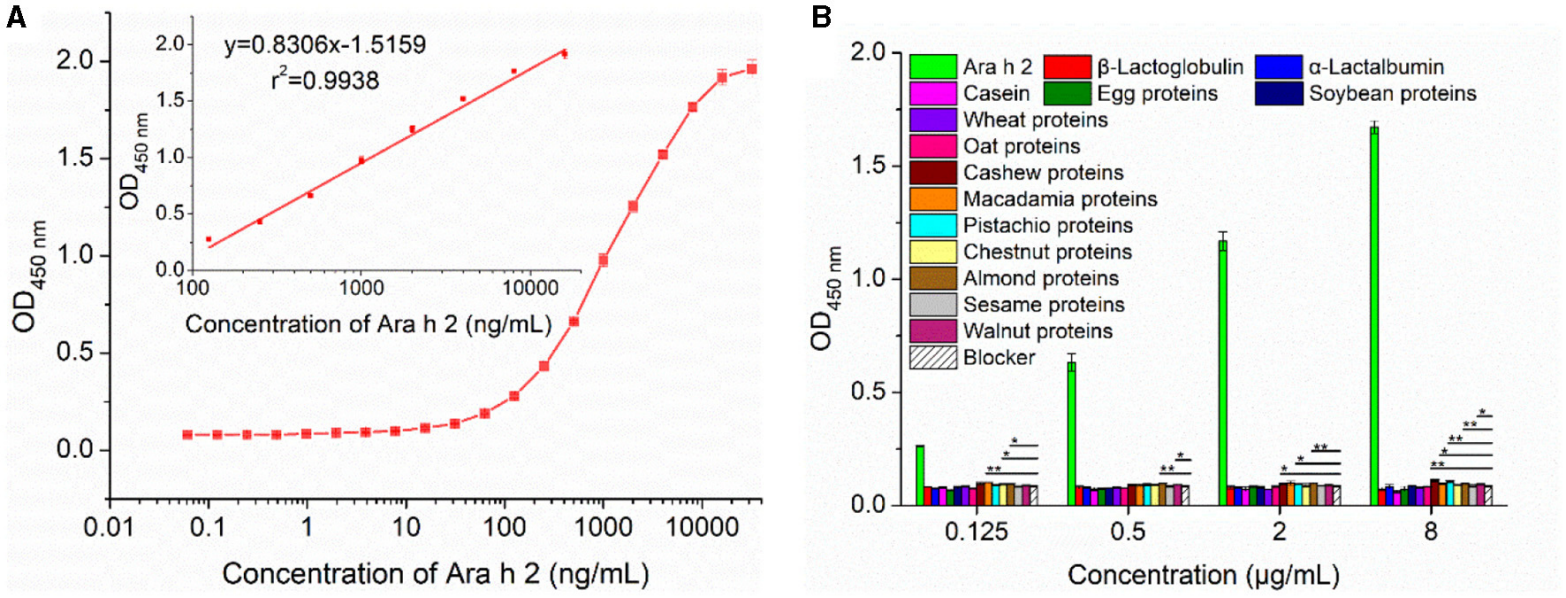
Performance analysis of the developed IgE-EsAb-based sELISA. **(A)** Calibration curves of IgE-EsAb-based sELISA for Ara h 2 detection. **(B)** Analysis of the specificity of the developed IgE-EsAb-based sELISA by testing the cross-reactivity with food allergens, and the blocking buffer serving as negative control. Data are expressed as mean ± SD (*n* = 3). Statistically significant at *p < 0.05 and **p < 0.01.

The sensitivity of the developed IgE-EsAbs-based sELISA was evaluated by assessment of the lowest detectable Ara h 2 concentration. The assay showed a LOD and LOQ of 0.98 ng/mL (0.98 ppb) and 3.91 ng/mL (3.91 ppb), respectively. Generation of a calibration curve (Figure 5A) further revealed a linear working range of 0.125–16 μg/mL (r^2^ = 0.9938).

Comparative analysis showed that the IgE-EsAbs-based sELISA has a lower LOD than most other analytical methods used for Ara h 2 detection (Supplementary Table S3). Most importantly, our IgE-EsAbs-based sELISA can specifically recognize IgE epitopes of Ara h 2, which makes it able to detect Ara h 2 allergenic residues and with the potential to measure Ara h 2 IgE-binding variations in processed foods (18, 30). As shown in Supplementary Table S3, the only analytical method that detects Ara h 2 IgE epitopes and with a significantly lower LOD is the rat basophilic leukemia (RBL-2H3) immune cell-based biosensing platform, with a LOD of 0.1 fmol/L (~0.002 ppb) (39). However, given that this sensor-based analytical technique requires cells culture, IgE antibodies to trigger an immunoreaction, and specialized knowledge, the IgE-EsAbs-based sELISA may be a more suitable method when lower costs and less complexity are desired.

#### 3.4.2 The accuracy, precision, and specificity of the IgE-EsAbs-based sELISA

Assay accuracy and precision were evaluated by assessment of intra-assay and inter-assay variation, using five replicates of Ara h 2 varying in concentration from 0.125 μg/mL to 16 μg/mL. The average bias of the intra-assay was 0.88%, and the mean RSDr and RSDR were 8.02% (4.13%−12.56%) and 10.68% (3.35%−16.50%), respectively (Supplementary Table S4). These results suggest that the IgE-EsAbs-based sELISA has high accuracy and precision.

Assay specificity was evaluated by analyzing the CR with various food allergens at an allergen concentration ranging from 0.125 μg/mL to 8 μg/mL. A minor CR was observed for proteins of cashew, macadamia, pistachio, almond, and walnut, but not for any of the other nine food allergens (Figure 5B). These results indicate that the IgE-EsAbs-based sELISA is applicable for Ara h 2 detection with high specificity.

### 3.5 The applicability of the IgE-EsAbs-based sELISA for Ara h 2 detection in food samples

To assess the suitability of the IgE-EsAbs-based sELISA for detection of Ara h 2 in samples with a complex matrix, recovery experiments were conducted using samples extracted from various foods. As shown in Table 1, Ara h 2 was detected in all tested peanut-containing food samples. Analysis of spiked food samples demonstrated recoveries ranging from 79.00% to 120.78%. These results suggest that the developed immunoassay is a suitable method for the detection of Ara h 2 in food samples.

**Table 1:** Detection and recovery analysis of Ara h 2 concentrations in (spiked) food samples (*n* = 3).

**Food sample**	**Ara h 2 spike concentration (mg/mL^c^ or mg/g^d^)**	**Detected concentration (mg/mL^c^ or mg/g^d^)**	**Recovery (%)**
Raw peanut extract^a^	0	1.71 ± 0.26	
	0.25	1.95 ± 0.01	96.25 ± 3.29
	2	4.12 ± 0.09	120.78 ± 4.52
Boiled peanut extract^a^	0	0.25 ± 0.01	
	0.25	0.53 ± 0.05	111.38 ± 20.99
	2	2.24 ± 0.40	99.55 ± 20.13
Roasted peanut-1 extract^a^	0	0.56 ± 0.10	
	0.25	0.78 ± 0.05	89.94 ± 18.78
	2	2.61 ± 0.24	102.72 ± 12.13
Roasted peanut-2 extract^a^	0	0.37 ± 0.05	
	0.25	0.57 ± 0.01	79.00 ± 3.07
	2	2.60 ± 0.19	111.44 ± 9.73
Fried peanut extract^a^	0	0.19 ± 0.03	
	0.25	0.46 ± 0.01	104.40 ± 2.05
	2	2.14 ± 0.15	97.46 ± 7.64
Beverage-1^b^	0	0.42 ± 0.04	
	0.25	0.64 ± 0.05	88.43 ± 21.09
	2	2.39 ± 0.45	98.59 ± 22.73
Beverage-2^b^	0	0.18 ± 0.01	
	0.25	0.38 ± 0.06	81.28 ± 23.53
	2	2.13 ± 0.30	97.75 ± 14.86
Cookie^b^	0	0.25 ± 0.02	
	0.25	0.47 ± 0.04	90.87 ± 14.28
	2	2.25 ± 0.03	100.26 ± 1.32
Bread^b^	0	Not detected	
	0.25	0.25 ± 0.02	98.25 ± 7.91
	2	2.11 ± 0.31	105.61 ± 15.70
Dry baked cake^b^	0	Not detected	
	0.25	0.23 ± 0.04	93.37 ± 16.75
	2	1.97 ± 0.27	98.26 ± 13.70

^a^These extracts contain protein exclusively sourced from peanut, at concentrations of 18.20 mg/mL for raw peanut extract, 2.72 mg/mL for boiled peanut extract, 4.32 mg/mL for roasted peanut-1 extract, 4.00 mg/mL for roasted peanut-2 extract, and 7.68 mg/mL for fried peanut extract. ^b^These extracts contain proteins from different sources, i.e., peanut and milk for beverage-1; peanut, oat, almond, hazelnut, and walnut for beverage-2; wheat, peanut, egg, almond, milk, and soybean for cookie; wheat, egg, and milk for bread; wheat and egg for dry baked cake. Concentrations of peanut protein in these extracts were not determined. ^c^Applicable to peanut extracts and beverages. ^d^Applicable to cookie, bread, and dry baked cake.

### 3.6 Validation of the IgE-EsAbs-based sELISA for measurement of Ara h 2 IgE-binding variations in food samples

**Figure 6:**
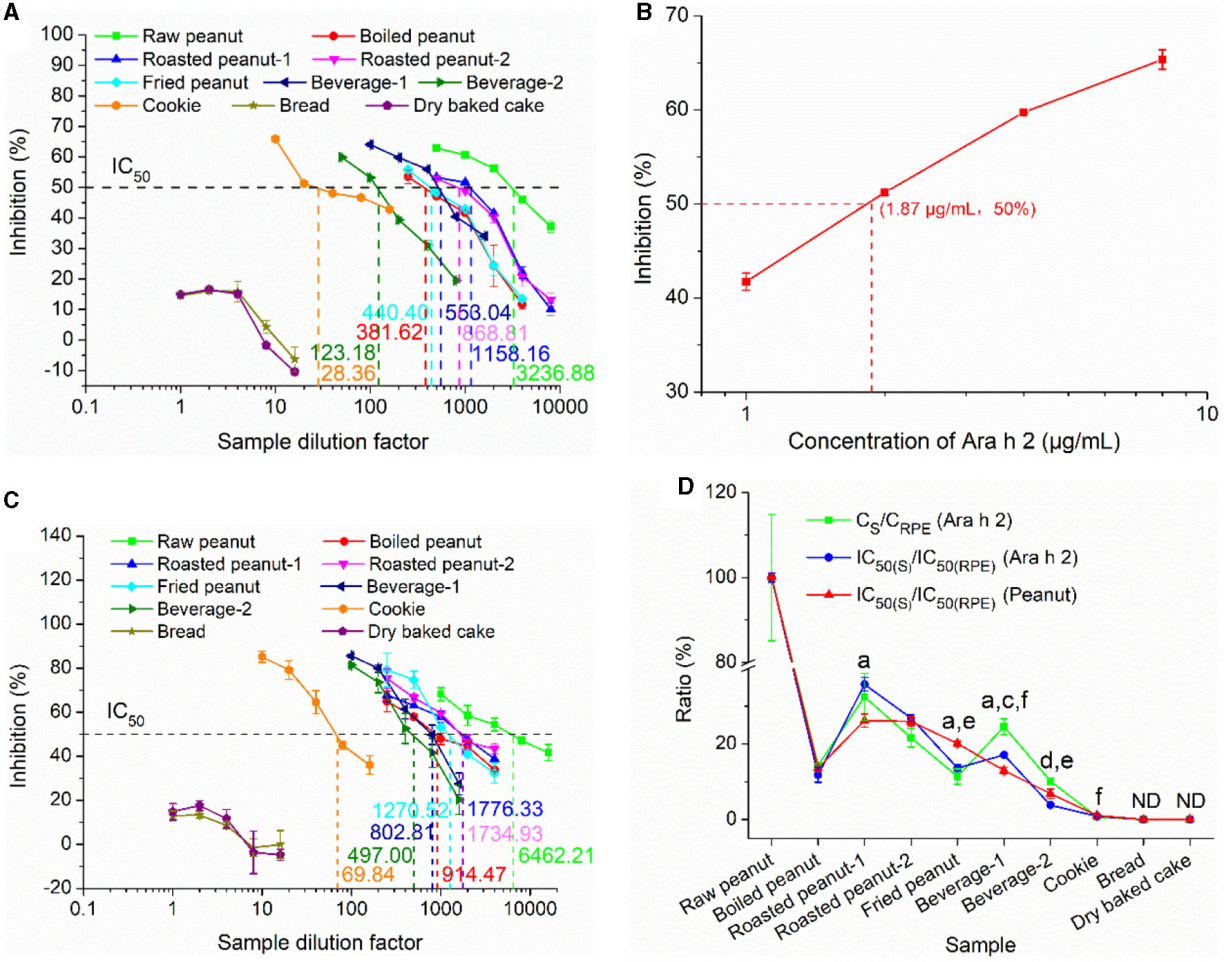
The capacity of the IgE-EsAb-based sELISA to measure peanut IgE-binding variations in foods assessed by cELISA using pooled sera from peanut-allergic individuals. **(A)** IgE-binding capacity of Ara h 2 in food samples. **(B)** IgE-binding capacity of the purified Ara h 2. **(C)** IgE-binding capacity of peanut in food samples. **(D)** Relation between Ara h 2 IgE epitope content, Ara h 2 IgE-binding capacity, and peanut IgE-binding capacity. Data are shown as mean ± SD (n = 3). C_S_ and C_RPE_ represent detected Ara h 2 IgE epitope contents in food samples and raw peanut extract, respectively. IC_50(S)_ and IC_50(RPE)_ denote the IgE-binding capacity of food samples and raw peanut extract, respectively. Statistically significant differences between Ara h 2 IgE-binding capacity and peanut IgE-binding capacity are indicated by a (*p* < 0.05) and b (*p* < 0.01), between Ara h 2 IgE epitope content and Ara h 2 IgE-binding capacity are indicated by c (*p* < 0.05) and d (*p* < 0.01), and between Ara h 2 IgE epitope content and peanut IgE-binding capacity are indicated by e (*p* < 0.05) and f (*p* < 0.01). ND, Not detected.

The IgE-EsAbs-based sELISA was tested for its capability to measure potential changes in IgE-immunoreactivity of Ara h 2 and peanut in various processed foods using sera IgE. The IgE-binding ability was quantified by competitive ELISA using pooled sera from peanut-allergic individuals. Ara h 2 immunoreactivity variations in different foods are illustrated in Figure 6A, the IC_50_ of RPE, boiled peanut extract, roasted peanut-1 extract, roasted peanut-2 extract, and fried peanut extract were found at dilution factors of 3236.88 (5.62 μg/mL protein), 381.62 (7.13 μg/mL protein), 1158.16 (3.73 μg/mL protein), 868.81 (4.60 μg/mL protein), and 440.40 (17.44 μg/mL protein), respectively. Taking into account that Ara h 2 comprises about 10% of total peanut proteins (40), the IC_50_ of Ara h 2 in these extracts corresponds to ~0.56 μg/mL, 0.71 μg/mL, 0.37 μg/mL, 0.46 μg/mL, and 1.74 μg/mL, respectively, which is lower than the IC_50_ of the purified Ara h 2 (1.87 μg/mL, Figure 6B). This deviation might be due to the presence of Ara h 6 and Ara h 7 in these extracts, which have a high homology with Ara h 2 (20), and may thus cross-react with patients' sera, resulting in lower IC_50_. Compared with the IC_50_ of RPE, the IC_50_ of roasted peanut-1 extract and roasted peanut-2 extract were lower, while the IC_50_ of boiled peanut extract and fried peanut extract were higher. This indicates that roasting enhances Ara h 2 IgE-immunoreactivity, while boiling/frying reduces it, which is consistent with other reports (6, 13, 41). The IC_50_ of beverage-1, beverage-2, and cookie were at dilution factors of 553.04, 123.18, and 28.36, respectively, indicating that the Ara h 2 IgE-immunoreactivity in these sample extracts was different. Finally, for bread and dry baked cake, slight inhibition was observed at dilution factors lower than 4, despite these foods being labeled to contain no peanuts. This slight inhibition may be explained by the possibility that the pooled sera used to assess the inhibition contained serum of an individual that was allergic to other food allergens alongside peanut, leading to a cross-reaction at low sample dilutions (42). Alternatively, this may be explained by the relatively high concentration (i.e., 2%) of Tween-20 present in the buffer used for protein extraction, which can suppress the antigen-antibody reaction (43).

The IgE-binding variations of peanut in different food samples as measured using sera IgE from peanut-allergic patients is shown in Figure 6C. The IC_50_ of RPE, boiled peanut extract, roasted peanut-1 extract, roasted peanut-2 extract, and fried peanut extract were observed at dilution factors of 6462.21 (2.82 μg/mL protein), 914.47 (2.97 μg/mL protein), 1776.33 (2.43 μg/mL protein), 1734.93 (2.31 μg/mL protein), and 1270.52 (6.04 μg/mL protein), respectively. These findings indicate that roasting enhances human IgE-immunoreactivity of peanut, while boiling/frying reduces it, which is consistent with the findings on Ara h 2 IgE-immunoreactivity described above and to those of previous reports (40, 44, 45), suggesting that Ara h 2 could serve as a useful biomarker for predicting IgE-binding changes of peanut. The IC_50_ of beverage-1, beverage-2, and cookie were observed at dilution factors of 802.81, 4497.0, and 69.84 respectively, indicating that the IgE-binding ability of peanut in these sample extracts was different. Finally, similar to the observations on Ara h 2 IgE-immunoreactivity described above, slight inhibition was observed for samples of bread and dry baked cake at low dilution factors (4 or lower; Figures 6A, C).

To validate the reliability of our developed sELISA in measuring IgE-binding variations of Ara h 2 and peanut in foods, the relationship between Ara h 2 IgE-binding ability and peanut IgE-binding ability, and the dose-effect relationship between the Ara h 2 IgE epitope content and Ara h 2 (or peanut) IgE-binding ability were established (Figure 6D). The detected Ara h 2 concentration (C_RPE_) and the IgE-binding ability of RPE [IC_50(RPE)_] were used as positive controls. Regarding the relationship between Ara h 2 IgE-binding ability (Figure 6D, blue line) and peanut IgE-binding ability (Figure 6D, red line), although significant differences were observed between the ratios of IC_50(roasted peanut-1, fried peanut, and beverage-1)_ to IC_50(RPE)_ (Ara h 2) and the ratios of IC_50(roasted peanut-1, fried peanut, and beverage-1)_ to IC_50(RPE)_ (peanut), they had the similar trend, except for beverage-1. These results further indicate that Ara h 2 can serve as a reliable marker for predicting peanut IgE-binding capacity. In addition, as indicated in the dose-effect relationship between the Ara h 2 IgE epitope content (Figure 6D, green line) and Ara h 2 (or peanut) IgE-binding ability (Figure 6D, blue or red line), only fried peanut, beverages, and cookie showed significant difference, but they had the similar trend, except for beverage-1. Therefore, these findings highlight that there is a good dose-effect relationship between the Ara h 2 IgE epitope content and Ara h 2 (or peanut) IgE-binding ability, indicating that the developed immunoassay can reliably reveal and measure potential changes in immunoreactivity of Ara h 2 and peanut in food samples and overcome the shortcomings of the IgE-binding capacity test, which depends heavily on the sera IgE (limited and variable) from peanut allergy patients (6, 13, 41).

In addition, the allergenicity of peanut allergens in food products can be established by basophils/mast cells degranulation and skin prick testing. Studies have shown that the results of IgE-binding experiments are usually in good agreement with these results obtained by basophils/mast cells degranulation assays or skin prick testing (13, 45, 46), which indicate that the IgE-binding capacity has the ability to preliminary predict potential peanut allergenicity (18). Therefore, the good dose-effect relationships obtained in this study suggest that our developed IgE-EsAbs-based sELISA could be used as a preliminary test to predict *in vitro* Ara h 2 and peanut potential allergenicity in processed foods. Also, a more complete validation should be performed in further study.

## 4 Conclusion

This study describes the development and validation of a novel IgE-EsAbs-based sELISA for detection of Ara h 2 and measurement of its immunoreactivity variations in foods. First, it was demonstrated that the monoclonal and polyclonal antibodies generated for use as capture and detection antibodies in the assay, respectively, could specifically recognize the target IgE epitope(s) of Ara h 2. Using these antibodies, the IgE-EsAbs-based sELISA exhibited high sensitivity (LOD = 0.98 ng/mL), specificity, and recovery (79.00%−120.78%) for Ara h 2 in food samples. Moreover, immunoreactivity changes of Ara h 2 in various food samples as tested by the IgE-EsAbs-based sELISA was consistent with that evaluated using sera IgE derived from peanut-allergic individuals. Together, these findings indicate that the developed immunoassay could serve as a sensitive, accurate, and relatively simplistic method for detecting Ara h 2 and measuring IgE-binding changes of Ara h 2 and peanut in food samples.

## Data availability statement

The original contributions presented in the study are included in the article/Supplementary material, further inquiries can be directed to the corresponding authors.

## Ethics statement

The studies involving humans were approved by the Peanut allergy patients' sera were provided by the First Affiliated Hospital of Gannan Medical University and approve by Gannan Medical University Research Ethics Committee (Reference number 2021105, 8/March/2021). Informed consent was obtained from all subjects involved in this study. The studies were conducted in accordance with the local legislation and institutional requirements. Written informed consent for participation in this study was provided by the participants' legal guardians/next of kin. The animal study was approved by the Gannan Medical University Animal Care Committee, under the guidelines of China Council for Animal Care (SYXK-Gan 2018-0004, China). The study was conducted in accordance with the local legislation and institutional requirements.

## Author contributions

YY: Data curation, Methodology, Writing—original draft. LL: Conceptualization, Resources, Supervision, Writing—review & editing. CL: Conceptualization, Writing—review & editing. YD: Methodology, Writing—review & editing. JL: Validation, Writing—review & editing. CS: Investigation, Writing—review & editing. YZ: Software, Writing—review & editing. JZ: Validation, Writing—review & editing. JW: Formal analysis, Writing—review & editing. AX: Visualization, Writing—review & editing. XL: Writing—review & editing. HC: Writing—review & editing. SH: Conceptualization, Data curation, Project administration, Supervision, Writing—review & editing.

## Supplementary material

Supplementary Figure S1 Mass spectrometry identification of Ara h 2 (including Ara h 2.01 and Ara h 2.02). **(A)** The amino acid sequence of Ara h 2.01. **(B–H)** Mass spectrum of Ara h 2.01**. (I)** The amino acid sequence of Ara h 2.02. **(J–P)** Mass spectrum of Ara h 2.02.

Supplementary Figure S2 The amino acid sequence (red letters) and gene sequence (black letters) of tAra h 2.

Supplementary Figure S3 The sequencing results of vector pET-28a(+)-tAra h 2. The tAra h 2 gene sequence was located between 225 and 725 bp.

Supplementary Table S1 Information of patients with peanut allergy.

Supplementary Table S2 Amino acid sequences of IgE epitopes and T cell epitope of Ara h 2.

Supplementary Table S3 Comparison of analytical methods for detection Ara h 2.

Supplementary Table S4 Evaluation of the accuracy and precision of the IgE-EsAb-based sELISA in the detection of Ara h 2.
