# Supplementary material for: A novel IgE epitope-specific antibodies-based sandwich ELISA for sensitive measurement of immunoreactivity changes of peanut allergen Ara h 2 in processed foods
Source: Front Nutr. 2024 Feb 19;11:1323553. doi: 10.3389/fnut.2024.1323553 (PMC10910080; doi:10.3389/fnut.2024.1323553)
Supplement: Supplementary Figure S1 — Mass spectrometry identification of Ara h 2 (including Ara h 2.01 and Ara h 2.02). (A) The amino acid sequence of Ara h 2.01. (B–H) Mass spectrum of Ara h 2.01. (I) The amino acid sequence of Ara h 2.02. (J–P) Mass spectrum of Ara h 2.02. [file Data_Sheet_1.docx]

**A novel IgE epitope-specific antibodies-based sandwich ELISA for sensitive measurement of immunoreactivity changes of peanut allergen Ara h 2 in** **processed foods**

Online Supplementary Material


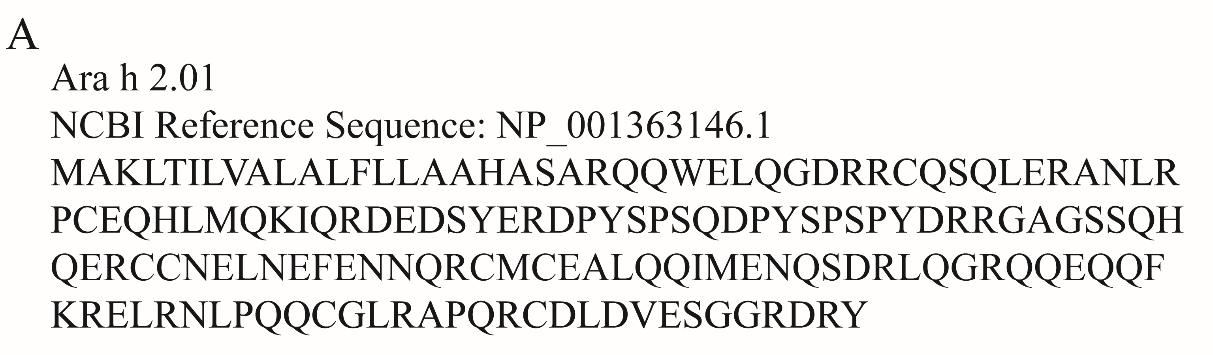


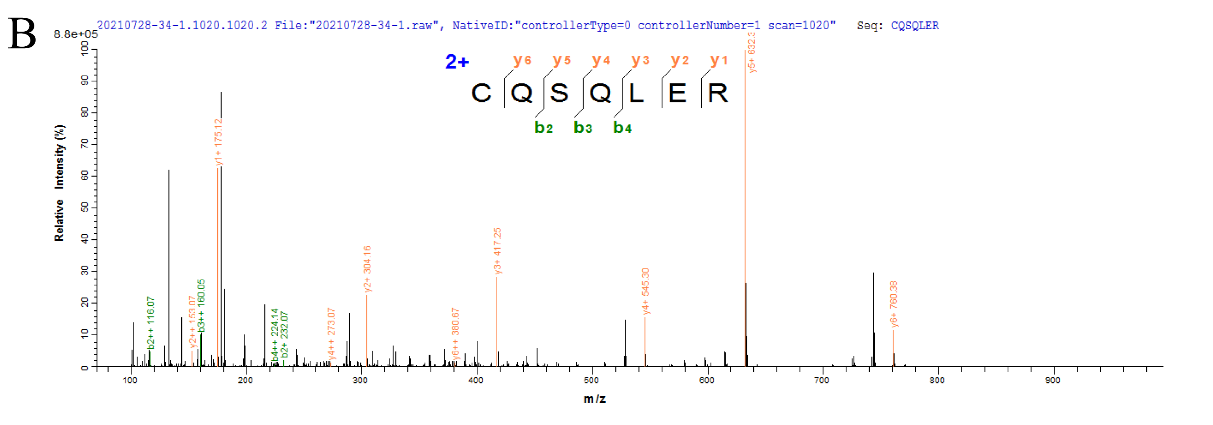


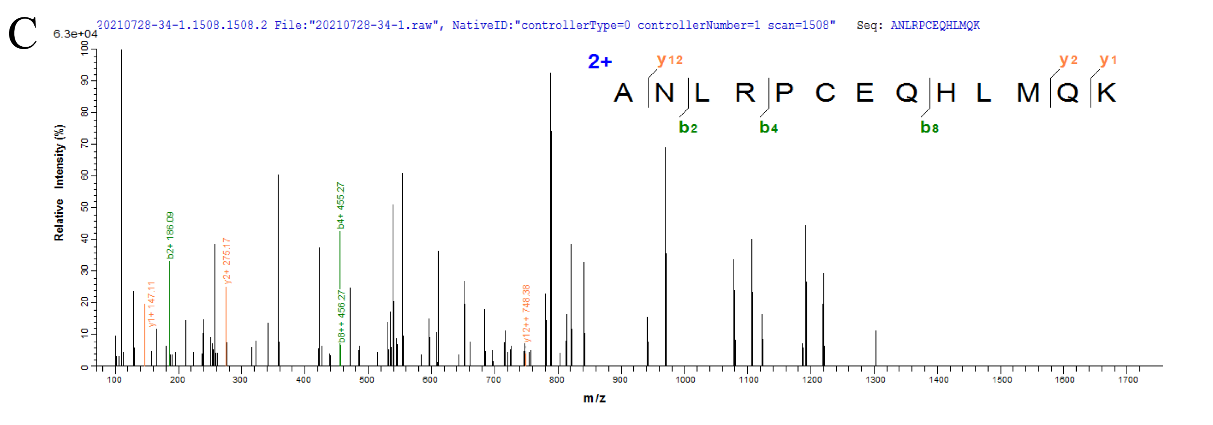


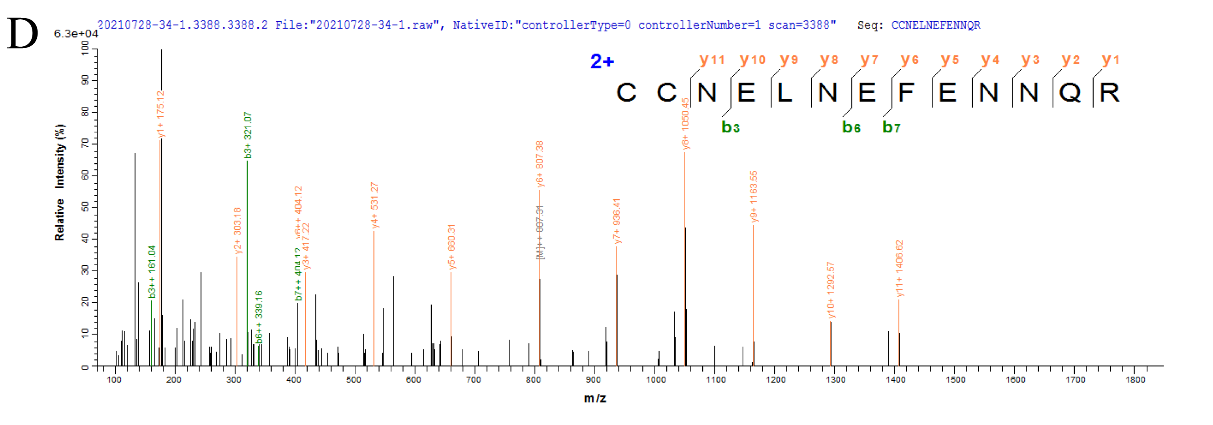


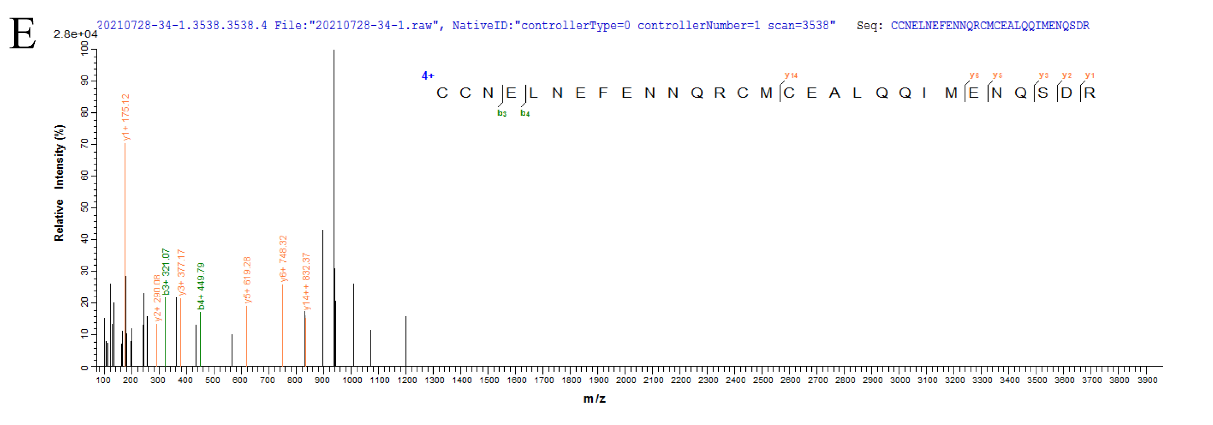


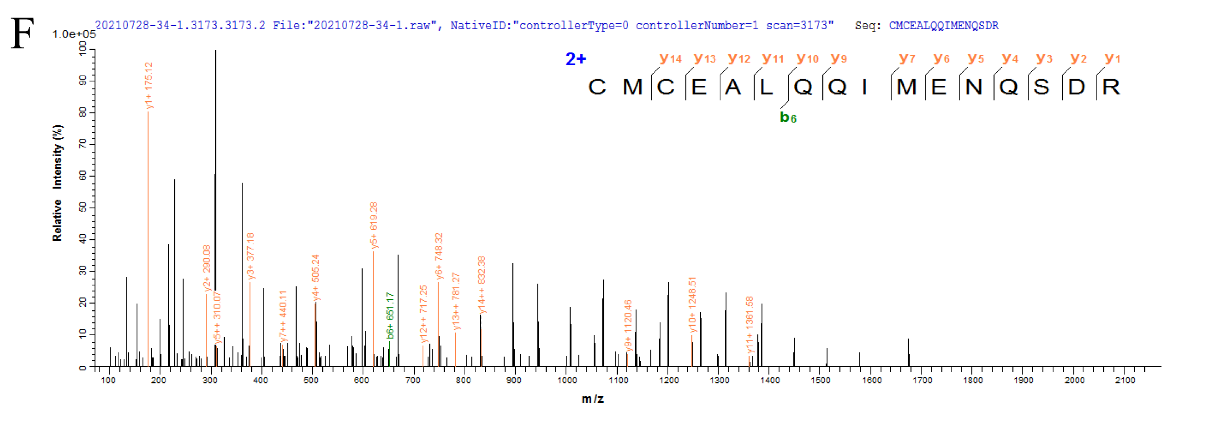


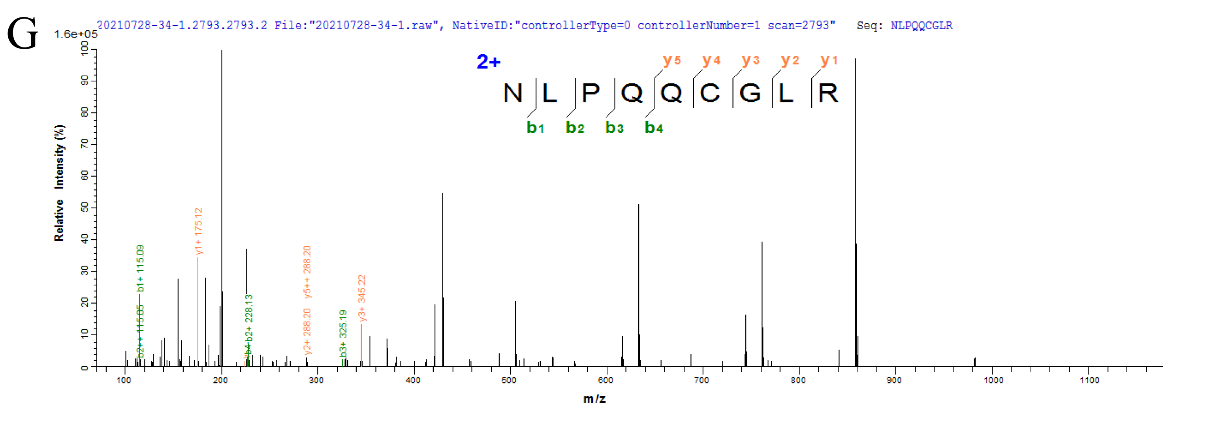


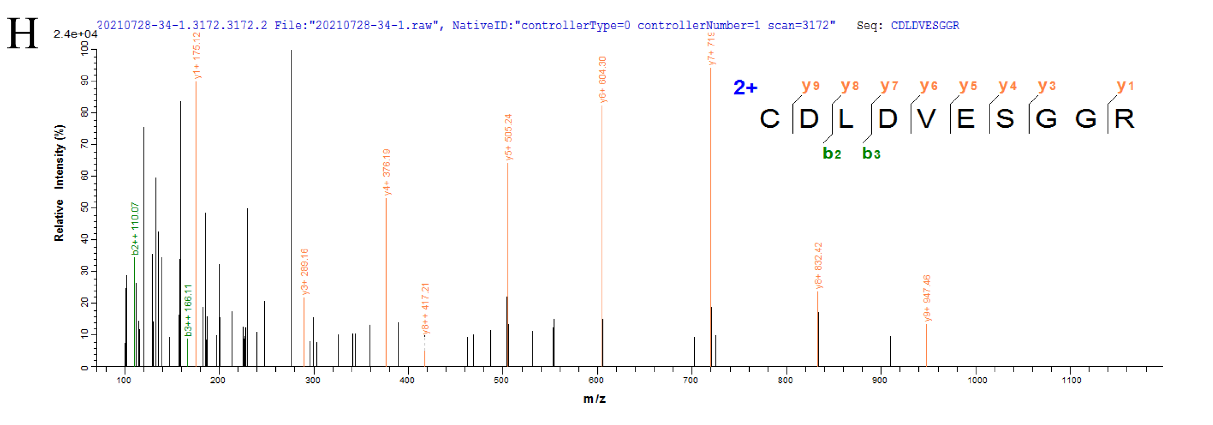


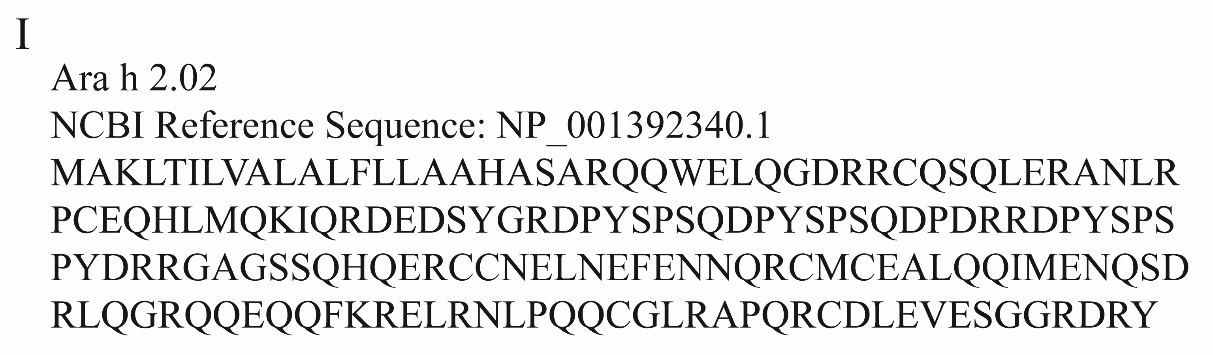


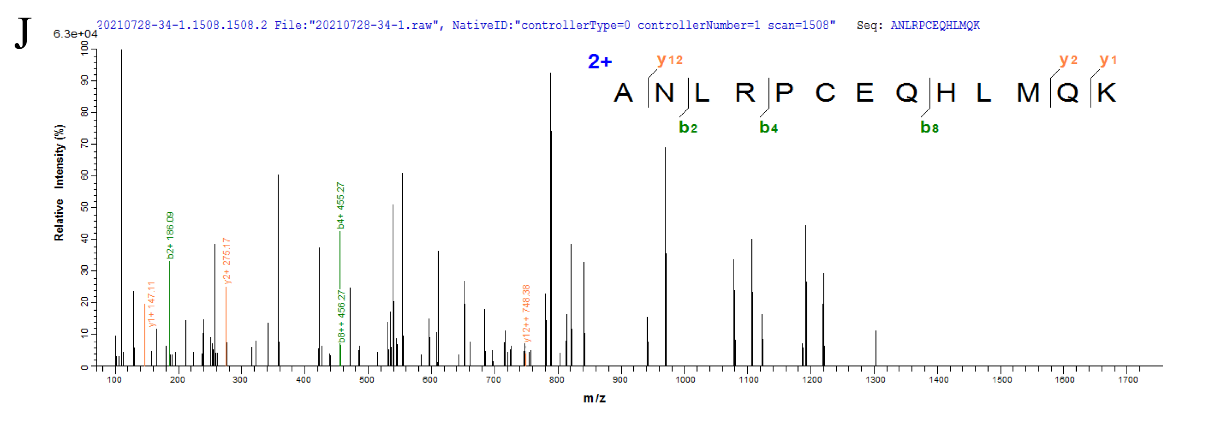


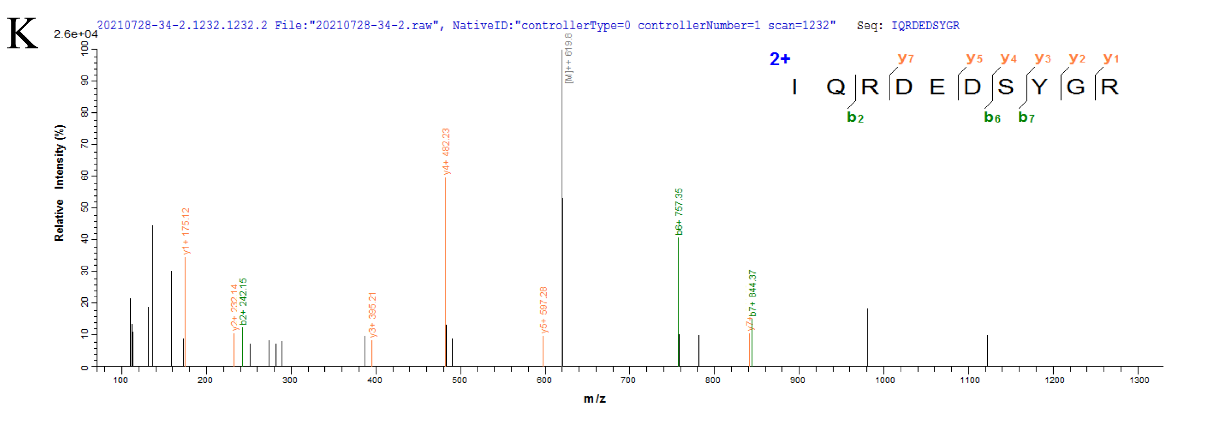


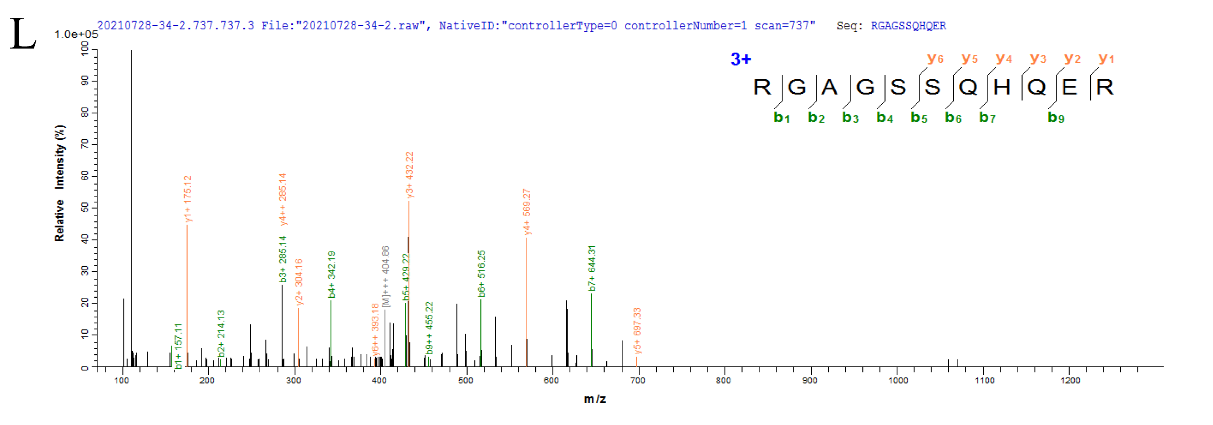


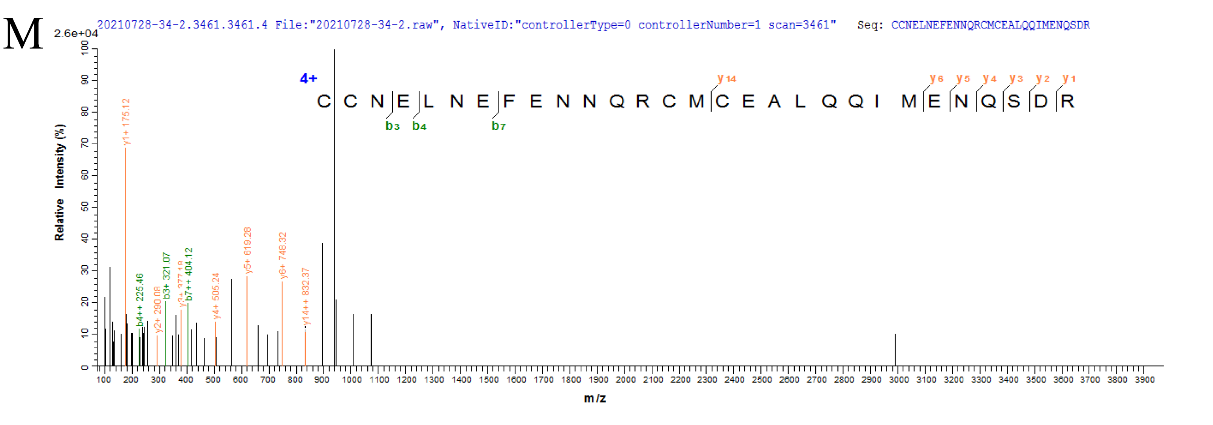


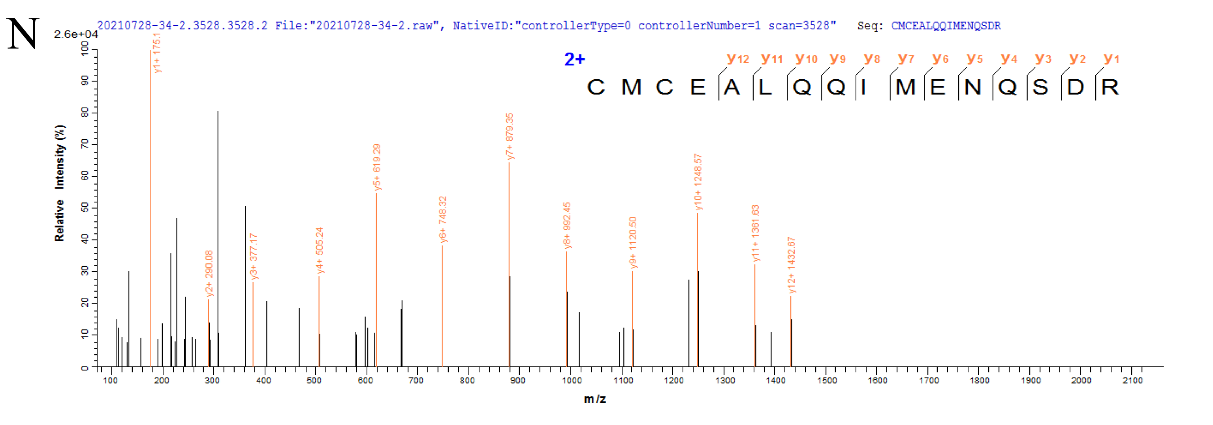


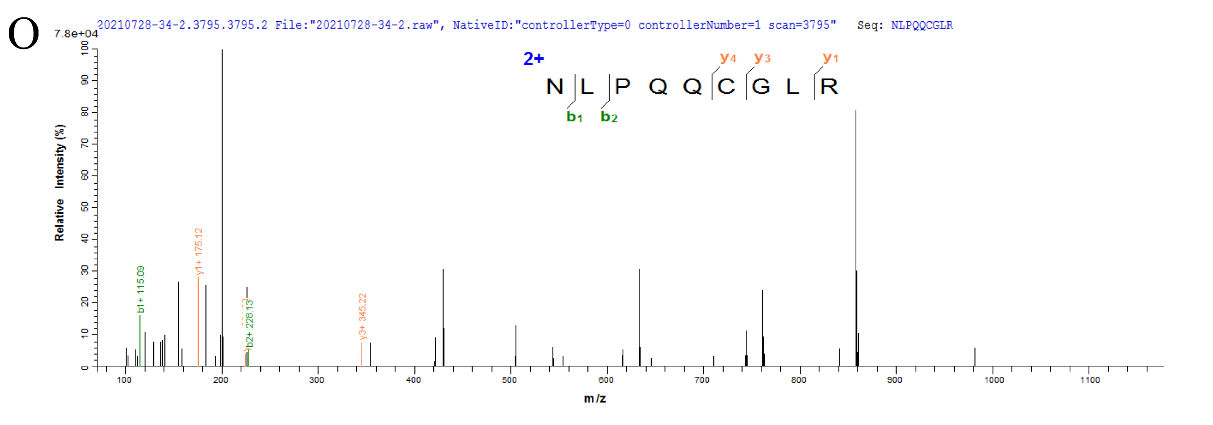


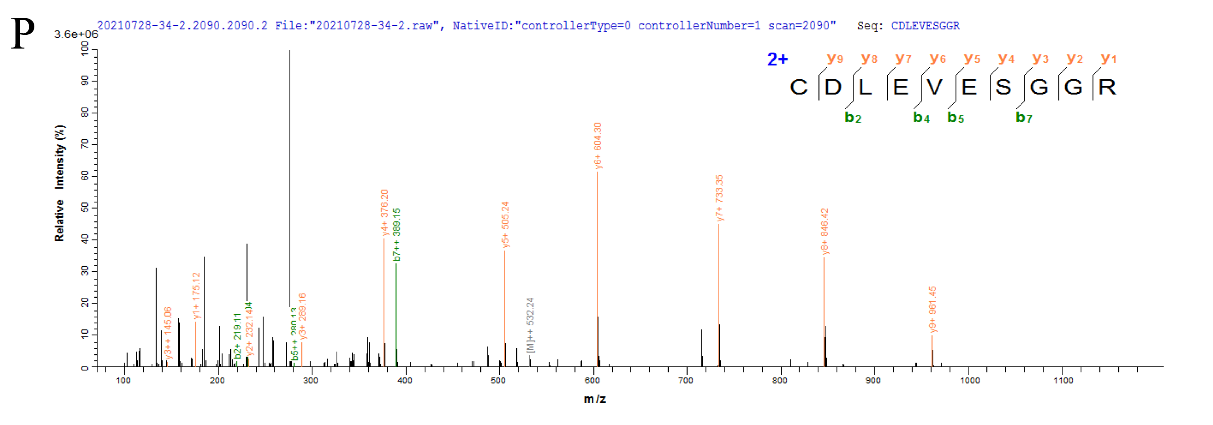


**SUPPLEMENTARY FIGURE S1** Mass spectrometry identification of the purified Ara h 2 (including Ara h 2.01 and Ara h 2.02). (**A**) The amino acid sequence of Ara h 2.01. (**B**–**H**) Mass spectrum of Ara h 2.01. (**I**) The amino acid sequence of Ara h 2.02. (**J**–**P**) Mass spectrum of Ara h 2.02.


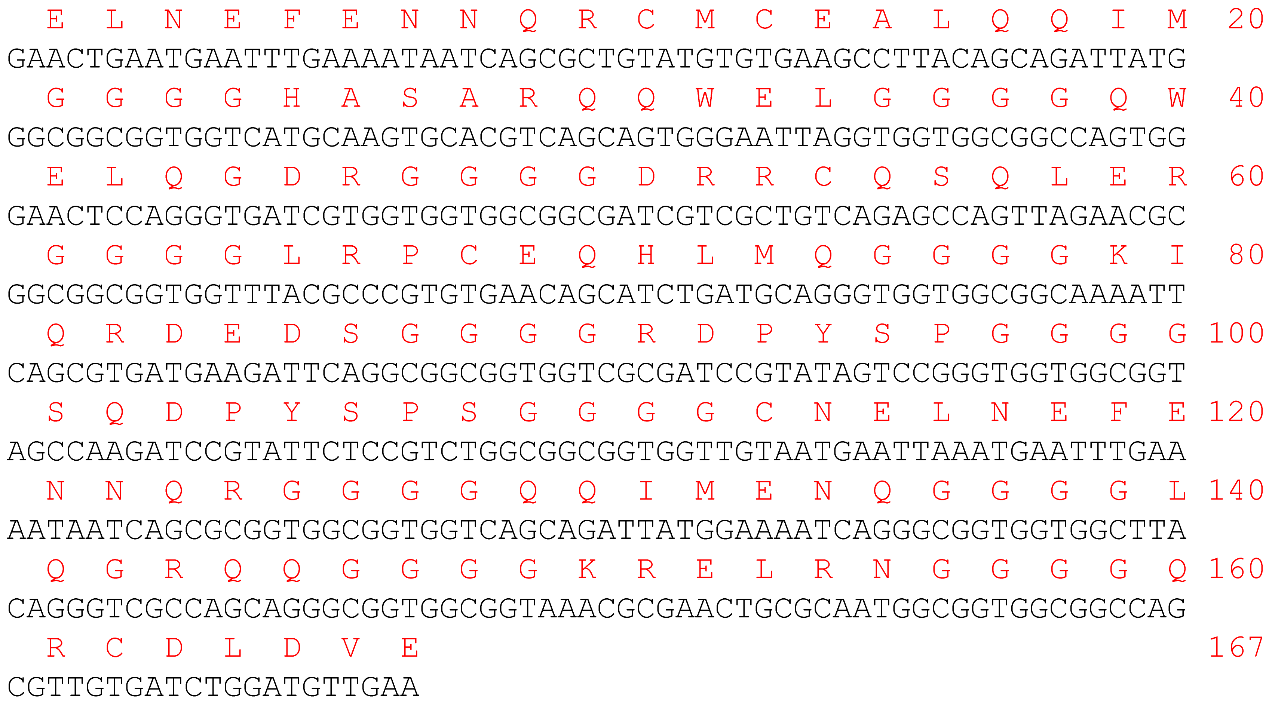


**SUPPLEMENTARY FIGURE S2** The amino acid sequence (red letters) and gene sequence (black letters) of tAra h 2.


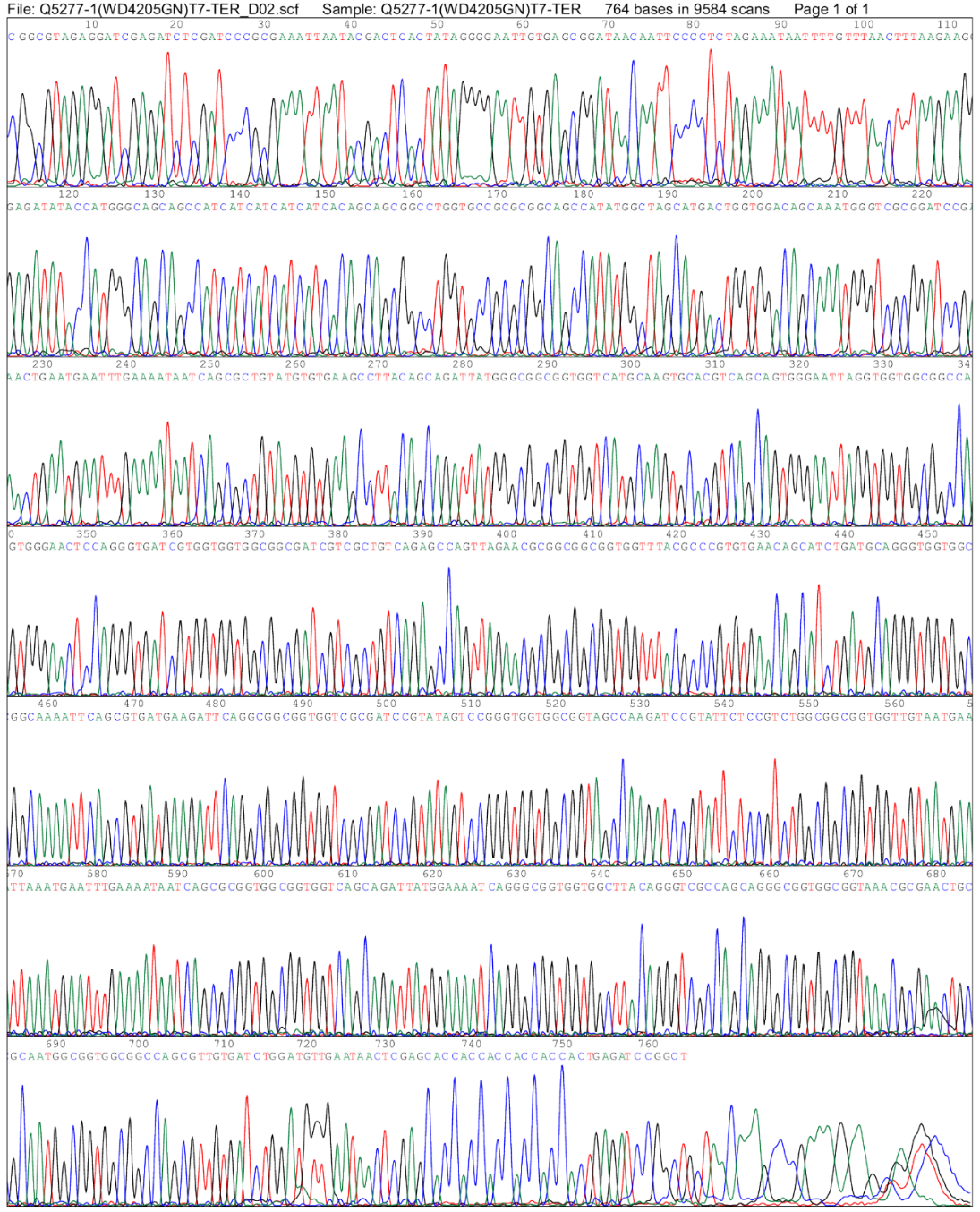


**SUPPLEMENTARY FIGURE S3** The sequencing results of vector pET-28a(+)-tAra h 2. The tAra h 2 gene sequence was located between 225 and 725 bp.

**SUPPLEMENTARY TABLE S1** Information of patients with peanut allergy.

| Number | Gender | Age | Clinical symptoms | Total IgE level (IU/mL) | Peanut specific IgE level (kU/L) |
| --- | --- | --- | --- | --- | --- |
| 1 | Female | 36 years old | urticaria | 132 | 0.7–3.5 |
| 2 | Male | 11 years old | urticaria | 73 | 0.35–0.7 |
| 3 | Female | 7 years old | papular urticaria | 149 | 0.35–0.7 |
| 4 | Male | 5 years old | atopic dermatitis | 485 | 0.35–0.7 |
| 5 | Female | 28 years old | chronic urticaria | 121 | 0.7–3.5 |
| 6 | Female | 43 years old | acute urticaria | 137 | 0.35–0.7 |
| 7 | Male | 2 years old | eczema | 315 | 0.35–0.7 |
| 8 | Female | 52 years old | bronchial asthma | not done | 0.35–0.7 |
| 9 | Male | 28 years old | unknown | 189 | 0.35–0.7 |
| 10 | Male | 2 years old | atopic dermatitis | 211 | 0.35–0.7 |
| 11 | Male | 5 years old | bronchial asthma | 291 | 0.35–0.7 |
| 12 | Female | 8 years old | pharyngitis, tic disorder | not done | 0.35–0.7 |
| 13 | Female | 5 months old | eczema | 96 | 0.7–3.5 |
| 14 | Female | 5 years old | [urticaria](javascript:;) | 233 | 0.7–3.5 |
| 15 | Male | 3 years old | atopic dermatitis | 277 | 0.35–0.7 |
| 16 | Female | 32 years old | chronic urticaria | 199 | 0.7–3.5 |
| 17 | Female | 8 months old | unknown | not done | 17.5–50 |

The total IgE level was determined by total IgE ELISA kit (EUROIMMUN, China).

The peanut specific IgE level was determined by EUROLINE ATOPY China (IgE) kit (EUROIMMUN, China).

**SUPPLEMENTARY TABLE S2** Amino acid sequences of IgE epitopes and T cell epitope of Ara h 2.

| Epitopes | [Abbreviation](javascript:void(0);) | Amino acid sequences |
| --- | --- | --- |
| IgE epitopes | B1 | AA18–27 (HASARQQWEL) |
|  | B2 | AA24–31 (QWELQGDR) |
|  | B3 | AA30–39 (DRRCQSQLER) |
|  | B4 | AA42–51 (LRPCEQHLMQ) |
|  | B5 | AA52–59 (KIQRDEDS) |
|  | B6 | AA62–67 (RDPYSP) |
|  | B7 | AA68–75 (SQDPYSPS） |
|  | B8 | AA92–103 (CNELNEFENNQR) |
|  | B9 | AA110–116 (QQIMENQ) |
|  | B10 | AA120–125 (LQGRQQ) |
|  | B11 | AA130–135 (KRELRN) |
|  | B12 | AA146–153 (QRCDLDVE) |
| T cell epitope | T | AA94–113 (ELNEFENNQRCMCEALQQIM) |

**SUPPLEMENTARY TABLE S3** Comparison of analytical methods for detection Ara h 2.

| Analytical methods | Specifically recognized epitope | LOD | Reference |
| --- | --- | --- | --- |
| LC-MS/MS | IgE epitopes | 5 ppm | [1] |
| Immunochromatographic | No | 1 ng/mL (1 ppb) | [2] |
| sELISA | No | 0.02 ng/mL (0.02 ppb) | [2] |
| sELISA | No | 5 ng/mL (5 ppb) | [3] |
| sELISA | No | 2 ng/mL (2 ppb) | [4] |
| sELISA | IgE epitopes | 1.3 ng/mL (1.3 ppb) | [5] |
| Electrochemistry | No | 0.2 nmol/L (~ 4 ppb) | [6] |
| Bioelectronic sensor | IgE epitopes | 0.1 fmol/L (~ 0.002 ppb) | [7] |
| sELISA | IgE epitopes | 0.98 ng/mL (0.98 ppb) | This work |

**SUPPLEMENTARY TABLE S4** Evaluation of the accuracy and precision of the IgE-EsAbs-based sELISA in the detection of Ara h 2.

| Ara h 2 (μg/mL) |  | Intra-assay*^a^* | | | |  | Inter-assay*^b^* | | |
| --- | --- | --- | --- | --- | --- | --- | --- | --- | --- |
|  |  | Mean±SD  (μg/mL) | Bias  (%) | RSDr*^c^* (%) | Replica  (n) |  | Mean±SD  (μg/mL) | RSDR*^d^* (%) | Replica  (n) |
| 0.125 |  | 0.15±0.008 | 20.00 | 5.33 | 5 |  | 0.14±0.011 | 7.86 | 5 |
| 0.25 |  | 0.23±0.016 | -8.00 | 6.96 | 5 |  | 0.23±0.012 | 5.22 | 5 |
| 0.5 |  | 0.43±0.041 | -14.00 | 9.53 | 5 |  | 0.44±0.065 | 14.77 | 5 |
| 1 |  | 0.95±0.079 | -5.00 | 8.32 | 5 |  | 1.03±0.17 | 16.50 | 5 |
| 2 |  | 1.99±0.25 | -0.50 | 12.56 | 5 |  | 2.00±0.31 | 15.50 | 5 |
| 4 |  | 4.33±0.36 | 8.25 | 8.31 | 5 |  | 4.29±0.51 | 11.89 | 5 |
| 8 |  | 8.88±0.80 | 11.00 | 9.01 | 5 |  | 8.92±0.92 | 10.31 | 5 |
| 16 |  | 15.24±0.63 | -4.75 | 4.13 | 5 |  | 15.54±0.52 | 3.35 | 5 |
|  |  | Mean | 0.88 | 8.02 |  |  | Mean | 10.68 |  |

*^a^*Assay was performed within one day (n=5).

*^b^*Assay was performed in 5 different days (n=3).

*^c^*Relative standard deviation of repeatability for intra-assay precision.

*^d^*Relative standard deviation of reproducibility for inter-assay precision.

**References**

1. Careri M, Costa A, Elviri L, Lagos JB, Mangia A, Terenghi M, Cereti A, Garoffo LP. Use of specific peptide biomarkers for quantitative confirmation of hidden allergenic peanut proteins Ara h 2 and Ara h 3/4 for food control by liquid chromatography-tandem mass spectrometry. *Anal Bioanal Chem.* (2007) 389:1901−7. doi: 10.1007/s00216-007-1595-2

2. Peng J, Song S, Liu L, Kuang H, Xu C. Development of sandwich ELISA and immunochromatographic strip for the detection of peanut allergen Ara h 2. *Food Anal Method.* (2015) 8:2605−11. doi: 10.1007/s12161-015-0163-1

3. Chen H, Zou Z, Tao A. A Quantitative method for detecting Ara h 2 by generation and utilization of monoclonal antibodies. *J Immunol Res.* (2018) 2018:4894705. doi: 10.1155/2018/4894705

4. Ng E, Nadeau KC, Wang S. Giant magnetoresistive sensor array for sensitive and specific multiplexed food allergen detection. *Biosens Bioelectron.* (2016) 80:359−65. doi: 10.1016/j.bios.2016.02.002

5. Schocker F, Scharf A, Kull S, Jappe U. Detection of the peanut allergens Ara h 2 and Ara h 6 in human breast milk: Development of 2 sensitive and specific sandwich ELISA assays. *Int Arch Allergy Immunol.* (2017) 174:17−25. doi: 10.1159/000479388

6. Zaitouna AJ, Lai R. An electrochemical peptide-based Ara h 2 antibody sensor fabricated on a nickel(II)-nitriloacetic acid self-assembled monolayer using a His-tagged peptide. *Anal Chim Acta.* (2014) 828:85−91. doi: 10.1016/j.aca.2014.04.033

7. Jeong JY, Kim SO, Bang S, Choi Y, Shin J, Choi D, Lee SE, Park TH, Hong S. Adaptive biosensing platform using immune cell-based nanovesicles for food allergen detection. *Biosens Bioelectron.* (2023) 222:114914. doi: 10.1016/j.bios.2022.114914
